# Cell wall composition and digestibility alterations in *Brachypodium distachyon* achieved through reduced expression of the UDP-arabinopyranose mutase

**DOI:** 10.3389/fpls.2015.00446

**Published:** 2015-06-17

**Authors:** David M. Rancour, Ronald D. Hatfield, Jane M. Marita, Nicholas A. Rohr, Robert J. Schmitz

**Affiliations:** ^1^U.S. Dairy Forage Research Center, United States Department of Agriculture – Agricultural Research Service, MadisonWI, USA; ^2^Department of Genetics, University of Georgia, AthensGA, USA

**Keywords:** plant cell wall, biomass, *Brachypodium distachyon*, grass, nucleotide–sugar, hydroxycinnamates

## Abstract

Nucleotide-activated sugars are essential substrates for plant cell-wall carbohydrate-polymer biosynthesis. The most prevalent grass cell wall (CW) sugars are glucose (Glc), xylose (Xyl), and arabinose (Ara). These sugars are biosynthetically related via the UDP–sugar interconversion pathway. We sought to target and generate UDP–sugar interconversion pathway transgenic *Brachypodium distachyon* lines resulting in CW carbohydrate composition changes with improved digestibility and normal plant stature. Both RNAi-mediated gene-suppression and constitutive gene-expression approaches were performed. CWs from 336 T_0_ transgenic plants with normal appearance were screened for complete carbohydrate composition. RNAi mutants of *BdRGP1*, a UDP-arabinopyranose mutase, resulted in large alterations in CW carbohydrate composition with significant decreases in CW Ara content but with minimal change in plant stature. Five independent *RNAi-RGP1* T_1_ plant lines were used for in-depth analysis of plant CWs. Real-time PCR analysis indicated that gene expression levels for *BdRGP1*, *BdRGP2*, and *BdRGP3* were reduced in *RNAi-RGP1* plants to 15–20% of controls. CW Ara content was reduced by 23–51% of control levels. No alterations in CW Xyl and Glc content were observed. Corresponding decreases in CW ferulic acid (FA) and ferulic acid-dimers (FA-dimers) were observed. Additionally, CW *p*-coumarates (*p*CA) were decreased. We demonstrate the CW *p*CA decrease corresponds to Ara-coupled *p*CA. Xylanase-mediated digestibility of *RNAi-RGP1 Brachypodium* CWs resulted in a near twofold increase of released total carbohydrate. However, cellulolytic hydrolysis of CW material was inhibited in leaves of *RNAi-RGP1* mutants. Our results indicate that targeted manipulation of UDP–sugar biosynthesis can result in biomass with substantially altered compositions and highlights the complex effect CW composition has on digestibility.

## Introduction

The plant CW is a complex mixture of carbohydrates, aromatic-compounds, and protein that is critical for plant form and function whilst comprising the largest source of renewable biomass on earth (Pauly and Keegstra, 2008). This lignocellulosic biomass material is used for food, feed, fiber, and energy-inputs. The acknowledgment of climate change and the need to support the growing global human population’s need for food and energy has put a high demand on technology development to maximize plant biomass production and utilization for sustainable and renewable sources of food, feed, fiber, and energy (FAO, 2003). Approaches to improve biomass quality, use-efficiency, and yields are needed.

The efficient extractability of CW carbohydrate is a major limitation in use-efficiency with many different proposed molecular solutions made to improve it (Hatfield et al., 1999c; McCann and Carpita, 2008; Pauly and Keegstra, 2008; Carroll and Somerville, 2009; Pauly and Keegstra, 2010; Jung et al., 2012; Doblin et al., 2014; Kalluri et al., 2014). Plant CW compositions are not uniform and vary according to many factors including species source, organ/tissue source, and developmental stage. The feasibility and efficacy of the proposed approaches may be plant species-dependent.

In monocots, the principle CW carbohydrate polymers are cellulose, β-glucans [(1,3;1,4)-β-D-glucans], and arabinoxylans (Carpita, 1996) with Glc, Xyl, and Ara dominating the CW carbohydrate profile (Pauly and Keegstra, 2008). The plant CW can be considered a metabolic sink for carbohydrate. Sugar biosynthesis and availability is critical to provide sufficient substrate for CW polysaccharide biosynthesis throughout plant growth and development.

Nucleotide–sugars are the principle carbohydrate-donor substrates for the glycosyltransferases involved in plant CW polysaccharide biosynthesis (Reiter, 2008; Bar-Peled and O’Neill, 2011). Biosynthesis of UDP-α-D-Xyl and UDP-β-L-Ara can be derived from a linear pathway initiating with UDP-α-D-Glc. The conversion of UDP-α-D-Glc to UDP-α-D-glucuronic acid (GlcA) by the UDP-α-D-Glc dehydrogenase (UGD) is the first committed step in the pathway (Tenhaken and Thulke, 1996). Subsequent steps are catalyzed by consecutive enzymes including: (a) UDP-α-D-GlcA decarboxylase (UXS) to make UDP-α-D-Xyl (Harper and Bar-Peled, 2002), (b) UDP-α-D-Xyl epimerase (UXE) to make UDP-β-L-Ara*p* (Burget et al., 2003), and (c) UDP-β-L-Ara*p* mutase (UAM; alternatively know as RGP or “reversibly glycosylated protein”) to make UDP-β-L-Ara*f* (Dhugga et al., 1997; Delgado et al., 1998; Konishi et al., 2007). A bifunctional pathway branch enzyme, UDP-α-D-apiose/UDP-α-D-Xyl synthase (AXS), exists that utilizes UDP-α-D-GlcA to make UDP-α-D-apiose (Api) and UDP-α-D-Xyl (Mølhøj et al., 2003). UDP-α-D-apiose is a critical component for rhamnogalacturonan II (RG-II) pectin biosynthesis. Phylogenetic analysis of representative family members has been performed (Yin et al., 2011) highlighting the conservation of this pathway in plants. Recent work has demonstrated that certain classes of UDP-α-D-Glc 4-epimerase are bifunctional and can epimerize UDP-α-D-Xyl to UDP-β-L-Ara*p* (Kotake et al., 2009).

Analysis of CWs from plants of mutants or altered expression of select genes encoding enzymes of this pathway has been performed in dicots including *Arabidopsis* UGD (Reboul et al., 2011; Behmüller et al., 2014), UXE (Burget et al., 2003), and UAM/RGP (Rautengarten et al., 2011), *Nicotiana benthamiana* AXS (Ahn et al., 2006), and alfalfa UGD (Samac et al., 2004). *In vivo* CW consequences of altered expression of this pathway have been limited in monocots and include such examples as maize UGD (Karkonen et al., 2005) and rice UAM/RGP (Konishi et al., 2011). In these presented cases, strong and negative consequences on CW formation and/or plant form and biomass production were observed.

Our hypothesis is that controlled manipulation of the biosynthesis of the nucleotide sugar substrates for CW polysaccharide biosynthesis could lead to changes in CW composition to improve CW digestibility but not affect plant growth and biomass production. We sought to explore manipulation of this UDP–sugar interconversion pathway in *Brachypodium distachyon*, a model C3 grass for forage grass research (Brkljacic et al., 2011; Mur et al., 2011; Philippe, 2011; Rancour et al., 2012; Girin et al., 2014). Targeting the UDP–sugar interconversion pathway in principle could lead directly to alterations in arabinoxylan and pectin structure, and in-directly into alterations in (1) CW crosslinking via hydroxycinnamates and (2) cellulose and/or β-glucans. For example, changes in the availability of UDP-GlcA, UDP-Xyl, and UDP-Ara would directly affect hemicellulose (Scheller and Ulvskov, 2010) and pectin (Atmodjo et al., 2013) biosynthesis but also indirectly affect FA-mediated crosslinking of CW components (Hatfield et al., 1999b; de O Buanafina, 2009). If the pathway is inhibited at the first committed step, then backup of UDP-Glc could lead to changes in CW cellulose and/or β-glucan content. However, little information regarding the relationship of (1) gene expression, (2) enzymatic-activity capacity for substrate biosynthesis, membrane transporters, and CW polysaccharide glycosyltransferases, and (3) overall metabolic flux is currently available in grasses to accurately predict the feasibility and outcome of these different scenarios on CW composition.

Therefore as a first step, the objectives of our study were to (1) characterize the expression of the UDP–sugar interconverting enzyme-encoding genes from *B. distachyon* and (2) use complementary RNAi and constitutive gene expression approaches to determine whether changes in gene expression would lead to alterations in CW composition without grossly affecting plant growth. We have identified RNAi-mutants in the *Brachypodium RGP1* which have reduced CW-bound Ara and hydroxycinnamates. RNAi-mutants exhibit increased xylan digestibility and decreased cellulose digestibility while not affecting plant stature. These data support the efficacy of a selection-scheme where mutant plants of near wild-type stature are screened for CW composition phenotypes. In addition, these data highlight the complexity of grass CW composition and ways to manipulate that complexity to improve digestibility.

## Materials and Methods

### General

All DNA primers (Supplementary Table S1) were synthesized by the DNA Synthesis Facility in the University of Wisconsin–Madison Biotechnology Center. All routine PCR (including colony PCR, A-tailing cDNA products, and transgenic plant screening) was performed using EconoTaq^®^ PLUS GREEN 2X master mix (Lucigen Corporation, Middleton, WI, USA). All DNA restriction and modifying reagents, unless noted otherwise, were obtained from New England Biolabs (Ipswich, MA, USA). All DNA sequencing was performed with ABI BigDye^TM^ Terminator reagents (Applied Biosystems-Life Technologies), Agencourt^TM^ CleanSEQ^TM^ (Agencourt Biosciences Corporation, Beverly, MA, USA) magnetic bead clean-up, and analyzed at the DNA Sequencing Facility in the University of Wisconsin–Madison Biotechnology Center.

### Vector Cloning

A modified, Gateway-cloning compatible *in planta* RNAi vector, based on the Ubi1p expression cassette for the pANDA series of monocot RNAi vectors (Miki and Shimamoto, 2004; Miki et al., 2005), was constructed essentially as described (Marita et al., 2014). One change to the previous construct was that the pPZP211 binary vector backbone (Hajdukiewicz et al., 1994) was used here in place of pPZP221b (Kang et al., 2001). The pPZP211 utilizes a CaMV 35S expression cassette controlling the expression of the neomycin phosphotransferase II plant selection marker.

A constitutive expression binary vector based on the Ub1 promoter-Gateway cassette from the pANDA mini vector (Miki and Shimamoto, 2004) was generated. The pANDA mini vector was digested with KpnI and EcoRV, overhang ends blunted with the NEB Rapid Blunting kit, and the resulting vector was ligated and transformed in to *ccdB* Survival^TM^ 2 T1^R^ *E. coli* (Invitrogen-Life Technologies). This processing resulted in a deletion of a ∼2.7 kb fragment containing the first Gateway cassette and the Gus linker of the pANDA mini RNAi hairpin cassette bringing the forward sense Gateway cassette 2 directly adjacent to the Ub1 promoter. The resulting plasmid was called *pANDA-OX mini*. The newly derived ZmUbi1p_rom_-Gateway-NosT cassette was PCR amplified (Finnzyme Phusion DNA polymerase) using primers RH12 and RH13 that introduced flanking FspI restriction sites. The PCR product was gel purified, phosphorylated with T4 polynucleotide kinase, and directly ligated into prepared pPZP211. The pPZP211 vector was prepared by restriction digest with EcoRI and blunting using T4 DNA polymerase. The presence and orientation of the insert was screened by restriction digest of isolated plasmid DNA using XhoI. The final empty binary vector, pPZP211-OX, was verified by DNA sequencing using primers RH3, RH4, RH5, RH6, RH10, RH11, RH14, and RH15.

### *Brachypodium distachyon* Nucleotide–Sugar Interconversion Enzyme-Encoding Gene Cloning

*Brachypodium distachyon* genes encoding homologs of UDP–sugar interconverting enzymes were identified via TBLASTN searches (Madden, 2013) of the *B. distachyon* Bd21 genome sequence (International Brachypodium Initiative, 2010) using protein sequences for *Arabidopsis*, rice, and maize homologs with verified function. Predicted protein sequences were obtained from NCBI. Protein sequence alignments and phylogenetic trees were generated using Lasergene MegAlign software (DNAstar, Madison, WI, USA) with the ClustalW multi-sequence alignment method using default parameters and the Gonnet-series protein-weight matrix. One thousand bootstrapping test trials were used for phylogenetic tree refinement.

DNA-free total RNA was isolated from frozen-ground *B. distachyon* Bd21-3 (Vogel and Hill, 2008) 7-days old whole seedlings and total aerial parts of 7-weeks old plants using the Spectrum^TM^ Plant Total RNA kit (Sigma–Aldrich, St. Louis, MO, USA) according to manufacturer’s protocol A. Total RNA yields and purity were calculated after spectrophotometer absorbance measurements at 260, 280, and 320 nm. First strand cDNA synthesis was performed using the GoScript^TM^ Reverse Transcription System (Promega, Madison, WI, USA) and a poly-T primer. One microgram of total RNA was used for cDNA synthesis reactions used for cloning.

PCR amplification of cDNAs for UGD1, UGD2, UGD3, UXS4, UXS6, UXE1, UXE2, UXE3, and AXS including coding sequence and partial 5^′^- and 3^′^-UTRs was performed using gene specific primers (Supplementary Table S2) and Phusion^TM^ high-fidelity DNA polymerase. Purified amplification products were ‘A-tailed’ and T-A sub-cloned into pGEM-T easy (Promega Corporation, Madison, WI, USA). The resulting clones were verified by DNA sequencing using primers RH8, and RH9 and DNA sequence analysis against the BD21 genome sequence was performed using DNAStar Lasergene software (DNAStar, Madison, WI, USA) (Supplementary Tables S3).

Sequence-verified T–A-cloned cDNAs were cloned into pENTR2B as follows. The pGEMT-easy cDNA vector DNA was digested with either NotI or EcoRI and blunted with the NEB Rapid Blunting Kit. The pENTR2B vector was double-digested with XmnI and EcoRV, de-phosphorylated with Antarctic Phosphatase (NEB), and T4 DNA ligase-mediated ligation reactions with added blunted-cDNAs were performed. Insert orientation was determined by PCR using pENTR2B-specific RH63 and 3^′^-cDNA-specific primers.

The cDNAs for UXS2, RGP1, RGP2, and RGP4 were PCR amplified using Phusion^TM^ high-fidelity DNA polymerase and gene-specific primers (Supplementary Table S2) and cloned into pENTR-D-TOPO using the pENTR^TM^ Directional TOPO^®^ Cloning Kit according to manufacturer’s instructions (Life Technologies). The resulting plasmids were validated by DNA sequencing using primers RH8 and RH9 (Supplementary Table S3).

Deletion mutant cDNAs used for construction of RNAi vectors were made by endonuclease-mediated removal of regions targeted by gene-specific qRT-PCR for endogenous transcript detection from pENTR-cDNA plasmids. Enzymes used and characteristics of regions removed and remaining are found in Supplementary Table S4.

To facilitate detection of constitutive expressed gene products, pENTR-cDNA plasmids for UGD1, UXS2, UXS6, UXE1, and AXS were modified to include a translatable C-terminal hemagglutinin protein peptide tag (HA-tag; YPYDVPDYA-stop). Plasmids were linearized by PCR using reverse orientation primers (see **Table 1**) and Phusion^TM^ high-fidelity DNA polymerase followed by DpnI-treatment of the resulting reaction mix with the products spin-column purified. Phosphorylated (5^′^) oligos (HA-forward and HA-reverse) encoding the HA-tag with a stop codon were self-annealed by mixing an equal molar ratio (10 μL of 100 μM each in TE), heated to 95°C in a thermal-cycler and allowed to cool to 4°C over 5 min. Annealed oligos were diluted with TE 10-fold and used directly in T4 DNA ligase-mediated ligation reactions with linearized plasmids (above). The resulting clones were screened by colony PCR using HA-tag and RH63 primers. The resulting plasmids were validated by single-pass DNA sequencing using primer RH63.

**Table 1:** Classification and nomenclature used for *Brachypodium distachyon* genes encoding UDP–sugar inter-conversion pathway enzymes.

Encoded enzyme	EC	Abbreviated name	Gene locus	*B. distachyon* nomenclature
UDP-glucose 6-dehydrogenase	1.1.1.22	UGD	Bradi1g08120	UGD1
			Bradi4g25140	UGD2
			Bradi1g10650	UGD3
UDP-xylose synthase (UDP-glucuronic acid decarboxylase)	4.1.1.35	UXS	Bradi1g66230	UXS1
			Bradi2g54380	UXS2
			Bradi2g11960	UXS3
			Bradi2g27870	UXS4
			Bradi1g18020	UXS5
			Bradi1g66440	UXS6
UDP-xylose 4-epimerase	5.1.3.5	UXE	Bradi5g21930	UXE1
			Bradi1g58080	UXE2
			Bradi3g14260	UXE3
UDP-arabinopyranose mutase (Reversibly Glycosylated Protein)	5.4.99.30	RGP (UAM)	Bradi1g15050	RGP1
			Bradi1g21990	RGP2
			Bradi2g50660	RGP3
			Bradi5g24850	RGP4
UDP-apiose/UDP-xylose synthase		AXS	Bradi2g61940	AXS
Expression control gene		UBC18	Bradi4g00660	UBC18

Gene-specific RNAi- and constitutive-expression binary vectors were generated using Gateway^®^ LR Clonase^TM^ II enzyme mix (Life Technologies) in miniature reactions. The reactions included ∼150 ng pENTR-cDNA, ∼150 ng destination vector, and Tris/EDTA (TE) pH 8.0 all to a volume of 5 μl. One microliter of LR Clonase^TM^ II enzyme mix was added and reactions were allowed to proceed for 1 h to overnight at room temperature. Reactions were terminated by adding 0.5 μL Proteinase K (supplied with LR Clonase kit), incubated for 15 min at 37°C, and then transformed into chemically competent DH5a *E. coli*. The resulting plasmids were verified by (1) endonuclease restriction digests and (2) DNA sequencing over recombination junctions using primers RH3, RH10, RH98, and RH99.

### RNA-seq Library Preparation, Sequencing, and Analysis

Total RNA was isolated as above from *B. distachyon* Bd21-3 plant tissues used for previous CW analysis (Rancour et al., 2012). RNA-seq libraries were constructed using Illumina TruSeq Stranded RNA LT Kit following the manufacturer’s instructions with limited modifications. The starting quantity of total RNA was adjusted to 1.3 μg, and all volumes were reduced to a third of the described quantity. Sequencing of libraries was performed up to 100 cycles. Image analysis and base calling were performed with the standard Illumina pipeline. Illumina HiSeq2500 output files in the FASTQ format were aligned to a SNP-substituted Bd21-3 *B. distachyon* reference genome using Bowtie version 2.1.0 (Langmead and Salzberg, 2012) and Tophat version 2.0.8b (Kim et al., 2013; flags = –F 0 – i 30 – M). Gene expression values were calculated using Cuﬄinks version 2.1.1 (flags = -u –library type fr-firststrand -b; Trapnell et al., 2013).

### Plant Transformation

The Bd21-3 accession was used for *Agrobacterium*-mediated plant transformation based on the method of Vogel and Hill (2008) with several modifications. Primary embryonic callus was cultured for 4 weeks on solidified *Brachypodium* callus induction medium (BCIM) [for 1 L: 4.43 g LS salts with vitamins, 30 g sucrose, 1 mL 0.6 mg mL^-1^ CuSO_4_, pH 5.8 with 0.1 M KOH, 2 g Gelzan (Caisson Labs, North Logan, UT, USA); after autoclaving add 0.5 mL of 5 mg mL^-1^ 2,4-dichlorophenoxyacetic acid (2,4-D)] and high-quality callus was fragmented and sub-cultured one time for one week prior to co-cultivation with *Agrobacterium*. The *Agrobacterium*-strain AGL1 (Lazo et al., 1991) was used for all experiments. *Agrobacterium* strains were streaked on MGL medium (for 1 L: 5 g tryptone, 2.5 g yeast extract, 5 g NaCl, 5 g mannitol, 0.1 g MgSO_4_, 0.25 g K_2_HPO_4_, 1.2 g glutamic acid, pH to 7.2 with 1 M NaOH, solidify with 15 g agar) containing selection (100 μg mL^-1^ carbinicillin and 100 μg mL^-1^ spectinomycin) and incubated at ∼28–30°C for 2 days prior to co-cultivation. *Agrobacterium* was scraped from MGL medium plates, suspended in sterile minimal liquid BCIM (for 1 L: 4.43 g LS salts with vitamins, 30 g sucrose, pH 5.8 with 0.1 M KOH) containing freshly prepared acetosyringone (stock solution of 200 mM in DMSO) at a final concentration of 200 μM and the OD_600nm_ adjusted to 0.6. Immediately prior to addition of callus, 10 μL of a 10% (w/v) Pluronic F68 (in water; sterile filtered) and 0.5 μL of 5 mg mL^-1^ 2,4-D per mL *Agrobacterium*-suspension was added. Callus was incubated 5 min in *Agrobacterium*-suspension, followed by decanting of excess solution and transfer of callus to sterile Whatman 3M filter paper disks in petri dishes. Excess *Agrobacterium*-containing solution was removed by two changes of filter paper disks and calli were allowed to air dry for ∼10 min (total time from start of co-cultivation to plate sealing is ∼15 min). Plates were capped, sealed with parafilm, and incubated ∼22°C in the dark (the plate stack was wrapped in Al foil). After 3 days, calli were transferred to modified BCIM selection medium [BCIM^par,mer^; for 1 L: 4.43 g LS salts with vitamins, 30 g sucrose, 1 mL 0.6 mg mL^-1^ CuSO_4_, pH 5.8 with 0.1 M KOH, 6 g Phytoblend (Caisson Labs), autoclaved, then 100 μg mL^-1^ paromomycin (*Phyto*Technology Laboratories, Shawnee Mission, KS, USA; Guerche et al., 1987; Torbert et al., 1995) and 25 μg mL^-1^ meropenem (I.V. veterinarian-grade; Ogawa et al., 2008) were added (both from 1000x filter-sterilized stocks in water stored at –20°C)], plates were sealed with parafilm and allowed to incubate for 1 week at 28°C in the dark. After 1 week, viable callus regions were transferred onto fresh BCIM^par,mer^ medium and grown for an additional 2–3 weeks at 28°C in the dark. Viable callus was transferred to *Brachypodium* plant regeneration selection medium (BRM^par,mer^; for 1 L: 4.43 g LS salts with vitamins, 30 g maltose, pH 5.8 with 0.1 M KOH, 6 g Phytoblend; autoclaved, then 200 μL of 1 mg mL^-1^ kinetin, 100 μg mL^-1^ paromomycin, and 25 μg mL^-1^ meropenem were added prior to solidifying). Plantlets were regenerated over 2–4 weeks of incubation at ∼24°C under a 14 h, ∼40 μmol photons s^-1^ m^-2^ (cool-white fluorescent light) day/10 h night cycle. Plantlets were transferred to solid MS medium (for 1 L: 4.42 g MS salts with vitamins, 30 g sucrose, pH to 5.7 with 0.1 M KOH, 6 g Phytoblend; autoclaved) in magenta boxes and allowed to form roots over 2–4 weeks of incubation at ∼24°C under a 14 h, ∼40 μmol photons s^-1^ m^-2^ (cool-white fluorescent light) day/10 h night cycle. Plants with roots were transplanted to soil and grown to maturity in a Conviron (Pembina, ND, USA) E15 growth chamber maintained at 24°C with a 20 h light-4 h dark light-cycle with an average cool-white fluorescent light photon flux of 180 μmol s^-1^ m^-2^.

### Transgenic Plant Screening

Leaf genomic DNA (gDNA) was isolated based on the procedure of Marita et al. (2014). Multiplexed PCR screening of plants for the neomycin phosphotransferase II plant-selection marker and a general gDNA marker, gRGP3, was performed using primers RH196, RH197, RH164, and RH177.

From select transgene-positive plants (BCHH developmental stage range 57–61; Hack et al., 1992; Hong et al., 2011; Rancour et al., 2012), ∼70 mg of fresh-weight leaf tissue was harvested, collected into a capped 2-mL bead-beater tubes containing two 4 mm acid-washed glass beads, and snap-frozen in liquid nitrogen. Frozen tissue was pulverized by bead-beating and CWs was isolated using a scaled-down procedure based on Rancour et al. (2012). Isolated CWs (∼5 mg) were subjected to scaled-down H_2_SO_4_-hydrolysis, alditol actetate derivatization, and GLC-FID analysis procedure based on Rancour et al. (2012). CW Glc, Xyl, and Ara content was determined, relative molar ratios were calculated, and all were used to identify plants with altered CW composition (Supplementary Table S5).

### Plant Growth and Bulk Tissue Harvest

All soil-grown plants were grown in environment-controlled growth chambers as described before (Rancour et al., 2012).

*RNAi-RGP1* T_1_ plants were grown to seed fill stage (BBCH 69-75) and then organs were harvested, sorted, frozen in liquid nitrogen, and stored at –80°C. The organs collected included (1) leaf blades and (2) leaf-sheath and stem. Organs of six plants from individual transgenic-events were pooled. All tissue was freezer-milled as described (Rancour et al., 2012).

### Quantitative Real-Time PCR Analysis

Primer design for nucleotide–sugar interconverting enzyme genes was performed with online software as indicated from either (1) Roche Universal ProbeLibrary Assay Design Center^1^ (site link as of September 10, 2013) using default parameters, (2) QuantPrime^2^ (Arvidsson et al., 2008) or (3) Primer-Blast software (Ye et al., 2012) at the NCBI. Primer sequences RH179 and RH180 for *Brachypodium* UBC18, an identified gene expression-control (Hong et al., 2008), were designed using the Roche software (above). Tissue- and plant line-specific first-strand cDNA was used for quantitative real-time PCR using either SYBR green master mix (Applied Biosystems, Carlsbad, CA, USA) or Bullseye EvaGreen qPCR 2X master mix with ROX (MidSci, St. Louis, MO, USA) based on previous methods (Sullivan, 2009; Marita et al., 2014). Reactions were run using an ABI 7300 Real-Time PCR System using the following conditions: 10 min denaturation at 95°C, 45 cycles of 95°C for 10 s and 58°C for 1.5 min, followed by amplification product dissociation analysis. Real-time PCR data was analyzed using the LinRegPCR method (Ramakers et al., 2003) and software available at http://www.hartfaalcentrum.nl/index.php?main$=$files&sub$=$LinRegPCR (as of July 23, 2013). N_0_ values for individual reactions run in triplicate were determined and arithmetic means were used to calculate relative expression ratios and SD for target gene to UBC18 expression for the indicated plant organs.

### Large-Scale CW Isolation

Starch-free CW preparations from frozen, homogenized *Brachypodium organs* were made as described (Rancour et al., 2012).

### Large-Scale CW Analysis

Total CW carbohydrate (uronosyls and neutral sugars) was determined using a scaled-down procedure based on Rancour et al. (2012). Isolated CWs (∼6 mg) were subjected to H_2_SO_4_-hydrolysis.

The sequential analysis of CW ester- and ether-linked phenolic moieties was performed as described (Grabber et al., 1995; Hatfield et al., 2009; Rancour et al., 2012; Marita et al., 2014). Approximately 20 mg of dried CW material per sample was used for analysis. FA dimers (diferulic acid, DFA) presented represent the sum of areas of identified peaks corresponding to 8-8^′^-DFA, 8-5^′^-DFA 8-*O*-4^′^-DFA, 8-5^′^-DFA (benzofuran), 5-5^′^-DFA, and 8-5^′^-DFA (decarboxylated). Ester- and ether-linked phenolics were identified and quantified as trimethylsilane derivatives (40 μL TMSI, Pierce and 10 μL pyridine) by GLC-FID (HP6890) on a ZB-5ms column (Phenomenex, Zebron 25 m × 0.25 mm, 0.25 μm film). The GLC conditions were: injector 315°C, detector 300°C, and a temperature program of 220°C 1 min, 4°C min^-1^ to 248°C held 1 min, followed by 30°C min^-1^ to 300°C before holding for 16 min. All GC temperature programs were run at 20 psi constant pressure and a split ratio 35:1. Periodic validation of sample peak identifications were performed by GLC-MS. CW preparations were analyzed in duplicate and data was compiled according to genotype and tissue source.

The CW lignin concentration was determined using the modified acetyl bromide method of Hatfield et al. as before (Hatfield et al., 1999a; Fukushima and Hatfield, 2001; Rancour et al., 2012).

Gel-state 2D NMR was performed as described (Marita et al., 2014).

### FA-Ara and pCA-Ara Analysis

Analysis of ferulate (FA)- and *p*-coumarate (*p*CA)-linked Ara was performed based on adaptation to the method presented in Marita et al. (2003). CW samples (∼10 mg) were hydrolyzed in 2 mL 0.1 M trifluoroacetic acid (TFA) for 1 h at 100°C. CW residues were separated from hydrolysates by centrifugation. Supernatants were removed and filtered through 1 μm glass–fiber (Acrodisc^®^ 25 mm) syringe filter into to new tubes. CW residues were washed two times with 2 mL dH_2_O, filtered, and pooled with the original supernatant. UV-Vis absorption spectra (of 1:10 dilution; scan 240–450 nm) were taken. The pooled soluble fractions were passed through conditioned and equilibrated C-18 columns (Supelco ENVI-18, 3 mL). Pooled unbound and column wash (with 0.033M TFA) material had UV-Vis spectra taken (as above) to verify binding of phenolics. Phenolic-containing compounds were eluted with 100% methanol. Internal standards of 2-hydroxycinnamic acid and 5-5-diferulate (50 μg each; stocks: 1 mg mL^-1^ in 95% ethanol) were added and samples dried with a stream of filtered air. Phenolic-Ara- and phenolic-compounds were identified and quantified as trimethylsilane derivatives [40 μL TMSI (Thermo-Fisher) and 10 μL pyridine; incubated 30 min at 55°C] using GLC-MS (Shimadzu GCMS-QP2010) with a ZB-1 column (Phenomenex; 30 m × 0.25 mm × 0.25 μm film). The GLC conditions were injector 315°C and a temperature program of 200°C for 1 min, 4°C min^-1^ to 248°C, hold 1 min at 248°C, 30°C min^-1^ to 300°C and hold for 16 min. Conditions were run at constant pressure of 16 psi. Sample split ratio was 100. The MS data collection was in negative mode with scan speed of 1666, event time of 0.5 s, *m/z* scan range of 50 to 750, and acquisition occurring from 1.55 to 31 min during the run. Quantitative determinations were made from integration of total ion-count chromatogram peaks with corresponding *m/z* content including FA-Ara-TMS (*614, 599*), *p*CA-Ara-TMS (*584, 569*), FA-TMS (*338*), *p*CA-TMS (*308*), and internal standards 2-OH-TMS (*308*) and FA-dimer-TMS (*616*). Standards of FA, *p*CA, and FA-Ara (Hatfield et al., 1991) were used to verify product identification. All samples were analyzed in duplicate.

### Cell Wall Digestibility

#### Xylanase-Treatment

Cell wall samples (∼5 mg) were suspended in buffer (30 mM citrate/NaOH pH 4.5, 0.01% (w/v) NaN_3_) containing 2 U ml^-1^ xylanase (*Thermomyces lanuginosus* endo-1,4-ß-xylanase; Sigma–Aldrich, St. Louis, MO, USA) for a total reaction volume of 0.5 mL. The reaction was incubated 24 h at 37°C. Particulate material was pelleted by centrifugation (10 min, 20K ×*g*, ∼24°C) and the debris-free supernatant was transferred to new tubes. Total sugar release was assayed in a microtiter-plate format according to Masuko et al. (2005) using Glc for a standard. Sample absorbance at 490 nm was measured using a Synergy HT Multi-Mode Microplate Reader (BioTek, Winooski, VT, USA). Reactions lacking the addition of CW were used for background absorbance correction. All digestions were preformed in triplicate with sugar assay analytical duplicates for digest products. Glc standards were measured in triplicate over a range of 0 to 3 mM (0, 0.75, 1.5, 2.25, and 3 mM) resulting in a linear response and an *r*^2^ value of 0.994.

#### Accelerase 1000-Treatment

Cell wall samples were subjected to Accelerase 1000 (Genencor) enzyme hydrolysis within the CW hydrolysis analytical platform of the Great Lakes Bioenergy Research Center (Michigan State University, East Lansing, MI, USA) based on Santoro et al. (2010). CW material was subjected to a 6.25 mM NaOH pretreatment before enzyme hydrolysis. Glc and pentose release was assessed according to facility protocols. Four CW samples per genotype were analyzed in quadruplicate for each sugar class (*n* = 16).

#### Calculations and Statistics

Calculations were performed using Microsoft Excel software. One-way ANOVA statistical analysis with a *post hoc* Tukey test (alpha of 0.05 or 0.01, as indicated) was performed using GraphPad Prism 5.0F software.

## Results

Using predicted amino acid sequences for known *Arabidopsis*, rice, and maize enzyme homologs (Delgado et al., 1998; Harper and Bar-Peled, 2002; Burget et al., 2003; Suzuki et al., 2003, 2004; Karkonen et al., 2005; Pattathil et al., 2005; Klinghammer and Tenhaken, 2007; Konishi et al., 2007; Rautengarten et al., 2011; Reboul et al., 2011; Yin et al., 2011), TBLASTN searches (Madden, 2013) of the *B. distachyon* Bd21 genome sequence (International Brachypodium Initiative, 2010) were performed to identify *Brachypodium* homologs. Putative candidate protein sequences were obtained and sequence alignments and phylogenetic analysis were performed for UGD (Supplementary Figure S2), UXS (Supplementary Figures S2 and S3), UXE (Supplementary Figure S4), RGP/UAM (Supplementary Figure S5), and AXS (Supplementary Figure S6). The results indicate the *Brachypodium* genome contains putative genes encoding at least three UGD, six UXS, three UXE, four RGP, and a single AXS gene homolog (**Table 1**).

**FIGURE 1:**
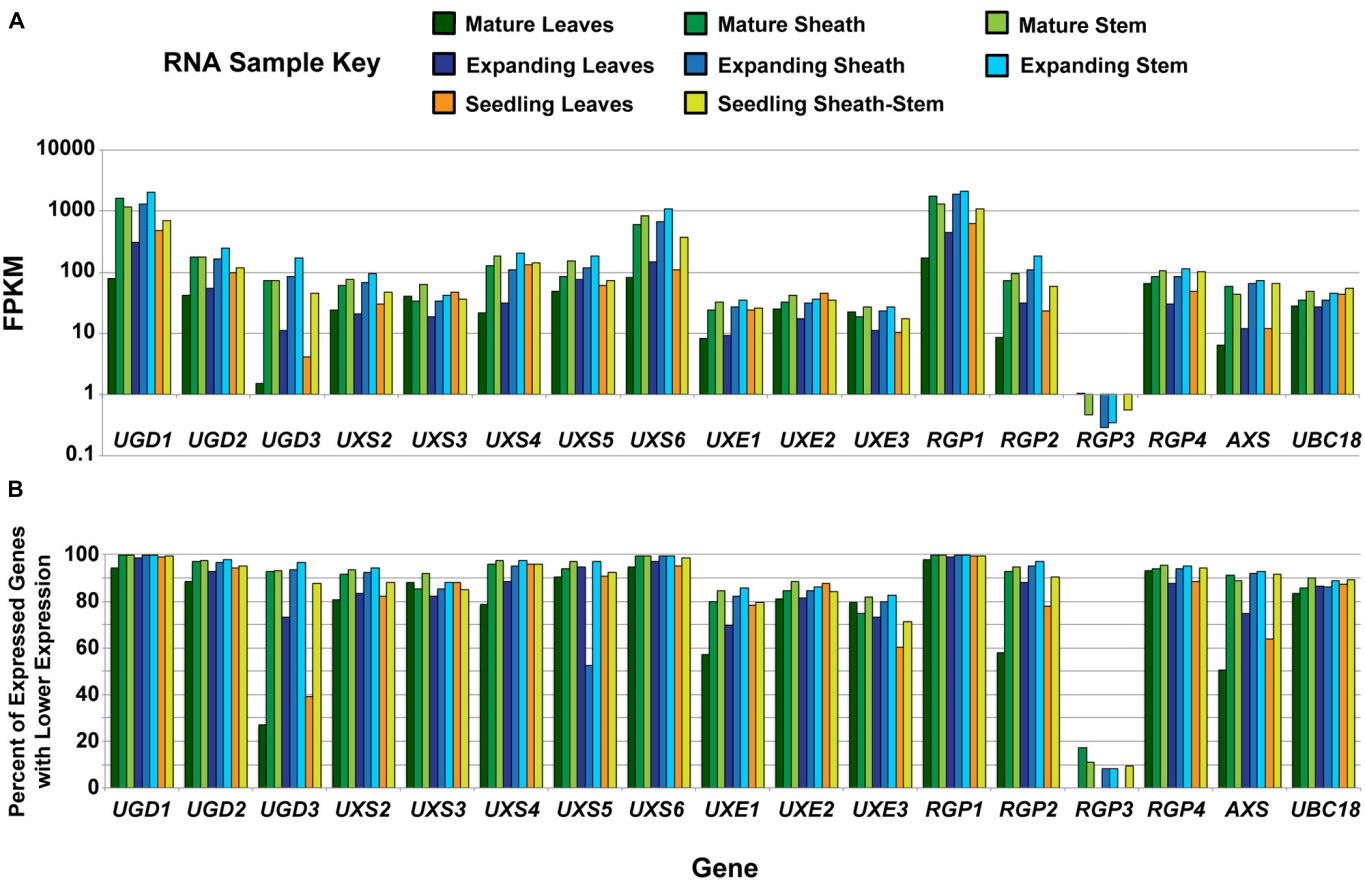
**Analysis of developmental expression of genes encoding UDP–sugar inter-conversion pathway enzymes in *Brachypodium distachyon*. (A)** Analysis of RNA from tissues previously characterized in Rancour et al. (2012) by RNA-seq for genes encoding UDP–sugar inter-conversion pathway enzymes and the real-time PCR amplification control (*UBC18*). The samples represent a developmental series of distinct tissues. A key is given. Data are log base 10 scale of fragments per kilobase of transcript per million fragments mapped (FPKM). **(B)** Expression ranking for genes encoding UDP–sugar inter-conversion pathway enzymes and the real-time PCR amplification control. Ranking values represent the percent of expressed genes with FPKM values lower than the indicated gene for the given tissue. For *RGP3*, expression in leaf blades at all developmental stages was not detected.

Cell wall composition analysis of *B. distachyon* aerial tissues at different stages of plant maturation was performed to provide a comparative bench-mark for using *Brachypodium* as a C3 forage grass model system (Rancour et al., 2012). RNA-seq was performed on RNA isolated from the eight Bd21-3 tissues analyzed in Rancour et al. (2012). The RNA-seq samples included (1) 12-day seedling leaf blades, (2) 12-day seedling pooled sheaths/stems, expanding stage (3) leaf blades, (4) sheaths and (5) stems, and mature stage (6) leaf blades, (7) sheaths, and (8) stems. Total gene expression profiles were determined to allow for comparative analysis with CW composition phenotypes and data for the UDP–sugar interconversion pathway members are presented (**Figure 1**). RNA-seq data for UDP–sugar interconversion pathway encoding genes indicated that *UXS1* (Bradi1g66230) was not expressed in the tissues analyzed. In addition, *RGP3* (Bradi2g50660) expression was present in sheath and stem samples but not leaf blade samples regardless of developmental stage.

Gene expression of *UGD1* (Bradi1g08120), *UXS6* (Bradi1g66440), and *RGP1* (Bradi1g15050) was consistently high throughout development and could be categorized in the top 1% of most-abundant genes expressed (**Figure 1B**). In addition, many of the remaining pathway encoding genes, including *UGD2*, *UGD3*, *UXS2*, *UXS4*, *UXS5*, *RGP2*, *RGP4*, and *AXS*, could be included in the top 10% of genes expressed in many samples analyzed. These data indicate that expression of genes encoding enzymes of the UDP–sugar interconversion pathway is relatively high in tissues that have extensive CW production and modification.

The UDP–sugar interconversion pathway is thought to provide the primary substrates for hemicellulose and pectin biosynthesis (Reiter, 2008; Bar-Peled and O’Neill, 2011; Yin et al., 2011; Atmodjo et al., 2013). We sought to test the hypothesis that targeted manipulation of expression of UDP–sugar interconversion pathway encoding genes could influence substrate availability, lead to changes in CW carbohydrate composition, and influence CW digestibility in grasses. Our goal was to change the CW characteristics with little affect on plant stature and development. To test this, cDNAs corresponding to a number of UDP–sugar interconversion pathway encoding genes were cloned (Supplementary Figure S7 and Table S3), constitutive expression (OX) and hairpin-RNAi constructs were made, and those binary constructs were used to transform *B. distachyon* Bd21-3 (**Table 2**). Large cDNA gene segments were used for the RNAi-constructs to take advantage of homologous regions between gene family members for potential whole-family suppression (Wesley et al., 2001). Additionally, cDNAs used for RNAi-constructs had select deletions corresponding to regions targeted by real-time PCR primers to allow for assessment of endogenous gene expression in a background where the RNAi-construct is being expressed (Supplementary Table S4). Transformation of the constructs resulted in numerous independent T_0_-lines that were screened (**Table 2**) for leaf CW Ara, Xyl, and Glc content (μmol g^-1^ CW) and alterations in Ara:Xyl and Xyl:Glc ratios (Supplementary Table S5). A threshold of two standard deviations from wild-type and empty-vector transgenic plants was used to mark the significance of the value changes observed. Based on these parameters, *RNAi-RGP1* plant lines had significant changes from wild-type values including decreased CW Ara content and decreases in Ara:Xyl ratios. Therefore, we sought to further characterize gene expression and the CW phenotype of the *Brachypodium RNAi-RGP1* plant lines.

**Table 2:** Summary of transgenic plants made in this study.

		TOTAL	CW CHO	
Target gene	Construct type	Events generated	Events screened	Plants screened
UGD1	OX	17	12	24
	RNAi	16	13	26
UGD2	RNAi	17	11	21
UXS2	OX	4	4	8
	RNAi	25	21	41
UXS4	RNAi	10	0	0
UXS6	OX	7	7	14
	RNAi	12	8	16
UXE1	OX	6	4	8
	RNAi	21	17	34
UXE2	RNAi	36	20	34
RGP1	RNAi	21	13	25
RGP2	RNAi	34	10	14
RGP4	RNAi	9	0	0
AXS	OX	4	4	8
	RNAi	43	27	45
Ubip::GusPlus	OX	21	3	6
Empty vector control	OX	6	6	12

**Total**		**309**	**180**	**336**

Total number of events generated per construct (TOTAL) and the number of subsequent events and plants per event screened for leaf CW carbohydrate (CW CHO) alterations.

**FIGURE 2:**
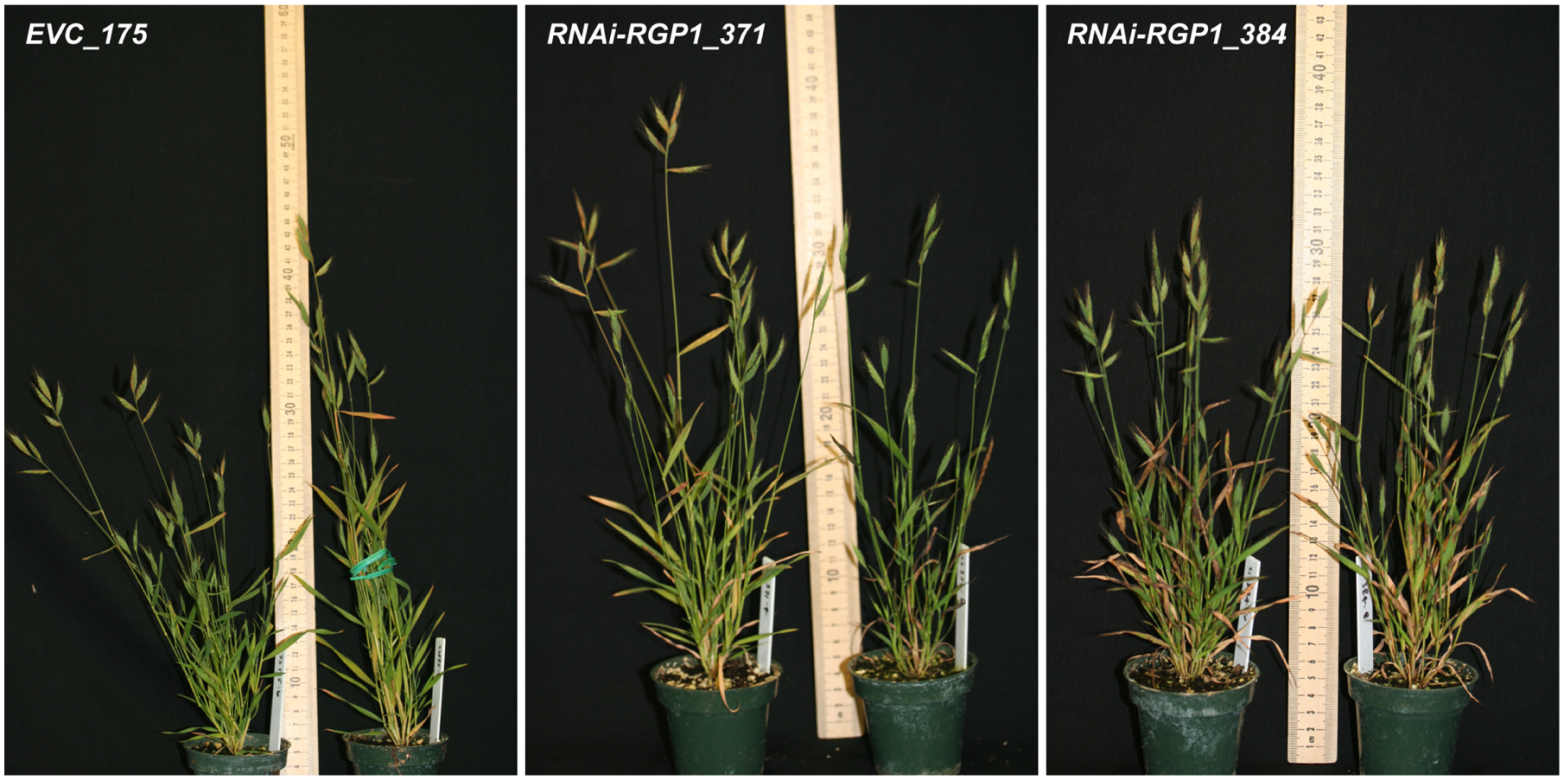
**Plant stature of T_1_ RNAi -UDP-β-L-Ara*p* mutase *B. distachyon* lines.** Representative plants from T_1_ transgenic empty-vector control line 175 (*EVC_175*) and *RNAi-RGP1* lines *371* (*RNAi-RGP1_371*) and *384* (*RNAi-RGP1_384*) at the time of harvest. Scale stick is in centimeters.

To ensure sufficient materials for complete analysis, aerial organs from independent-event T_1_-plants for five *RNAi-RGP1* (*RNAi-RGP1_371*, *373*, *382*, *384*, and *393*) and two empty-vector control (*EVC_173* and *175*) lines were harvested from six plants each, pooled, and processed in parallel. In parallel, wild-type Bd21-3 plants were grown and harvested at the same time as the T_1_ transgenic plants. Leaf blades (leaf) and leaf sheaths and stems (sheath/stem) were harvested, pooled according to plant-line, and used for subsequent analysis. Images of representative plants at harvest were taken (**Figure 2**). The morphology of RNAi-lines was comparable to empty vector control lines. The stature of *RNAi-RGP1*_*384* and *393* lines were modestly shorter and more uniform. For example, stem lengths were 24.8 ± 5.4 cm (mean ± SD; *n* = 14) for EVC_175, 22.5 ± 3.6 cm (*n* = 13) for *RNAi-RGP1_371*, and 20.0 ± 1.6 cm (*n* = 16) for *RNAi-RGP1_384*. ANOVA analysis (*post hoc* Tukey test; alpha = 0.05) indicated the difference between EVC_175 and *RNAi-RGP1_384* was statistically significant with a height reduction of 19%. Yields of CW from fresh weight tissue were comparable between control and *RNAi-RGP1* mutant lines (Supplementary Table S6).

### Cell Wall Carbohydrates

**FIGURE 3:**
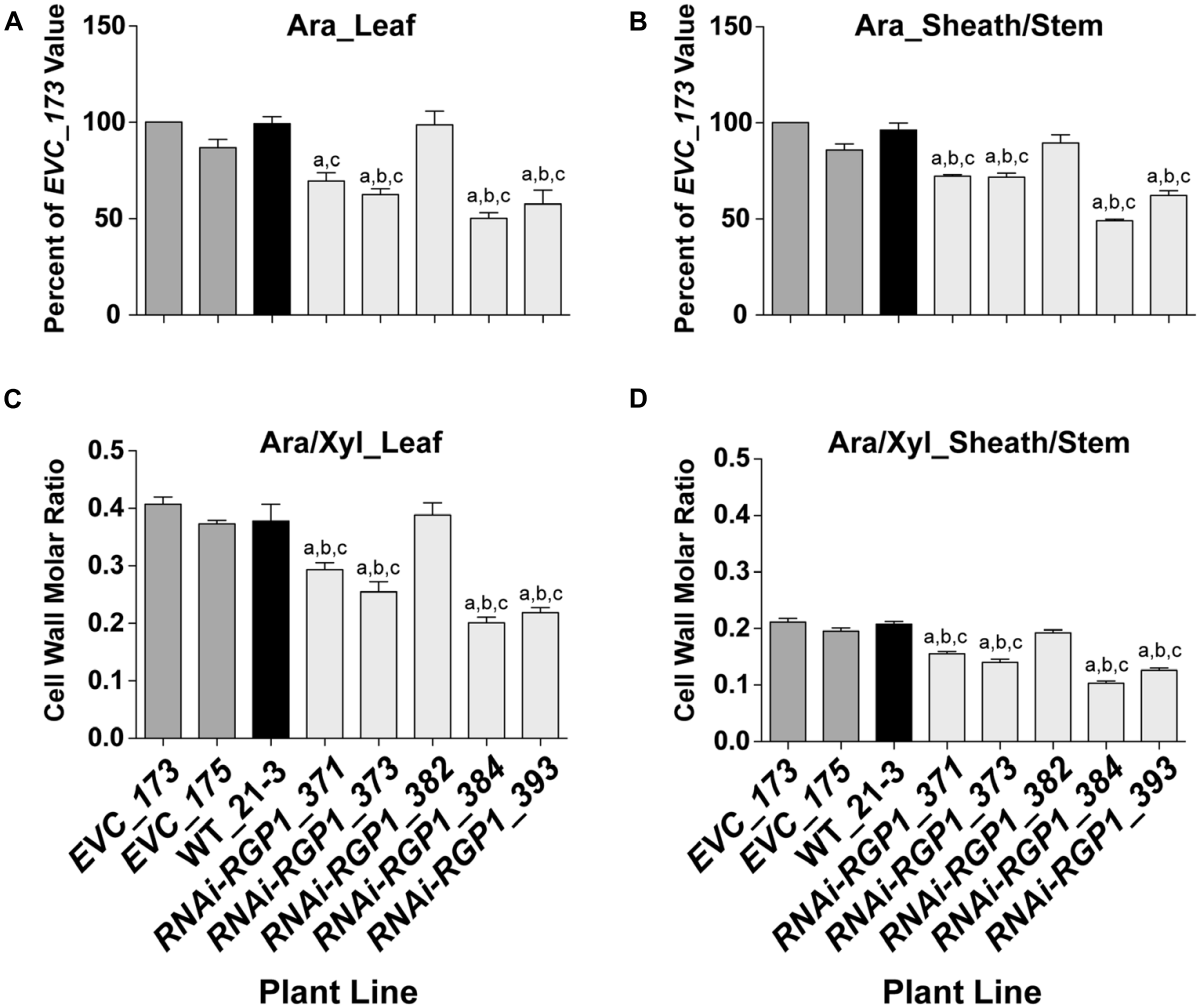
**Decreased CW Ara content in RNAi -UDP-β-L-Ara*p* mutase *B. distachyon* lines. (A)** Leaf blade and **(B)** leaf sheath/stem normalized CW Ara content of empty-vector control lines (*173* and *175*), wild-type Bd21-3 (WT_21-3), and *RNAi-RGP1* lines (*371*, *373*, *382*, *384*, and *393*) (left to right). All values were normalized with *EVC_173* set to 100%. The leaf and sheath/stem *EVC_173* values (mean ± SEM; *n* = 4) were 341.5 ± 25.4 and 247.9 ± 6.8 μmol Ara g^-1^ CW, respectively. **(C)** Leaf blade and **(D)** leaf sheath/stem CW molar ratios of Ara to Xyl of empty-vector control lines (*EVC_173* and *175*), wild-type Bd21-3 (WT_21-3), and *RNAi-RGP1* lines (*371*, *373*, *382*, *384*, and *393*). Error bars indicate SEM. ^a,b,c^ significantly different from *EVC_173*, *EVC_175*, and WT_21-3 values, respectively (ANOVA with *post hoc* Tukey test, alpha = 0.05).

To verify that the CW carbohydrate screening results were heritable, total CW sugars were analyzed from isolated CWs of leaf and sheath/stem fractions. CWs from leaf (**Figure 3A**) and sheath/stem (**Figure 3B**) both exhibited statistically significant decreases in Ara content in four of the five *RNAi-RGP1* lines when compared to empty vector control lines and wild-type Bd21-3 (WT) CWs. The reductions ranged from 27 to 51% of *EVC*_173 levels based on numerical mean values. *RNAi-RGP1* lines *384* and *393* exhibited the greatest reduction in CW Ara. The CW Ara content of *EVC* lines and wild-type plants were not statistically different. CW Glc, Xyl, and uronosyl content did not deviate from *EVC* and WT values (Supplementary Table S7).

The CW molar ratio of Ara to Xyl in grasses is used as an indicator of the relative substitution of the β-1,4-xylan backbone with Ara subunits. Given that the CW Glc and Xyl did not change in *RNAi-RGP1* mutants, the change in leaf (**Figure 3C**) and sheath/stem (**Figure 3D**) CW molar ratios of Ara to Xyl reflected the decrease in total CW Ara content. These results indicate that in select *RNAi-RGP1* mutant *Brachypodium* lines, CW Ara is reduced up to 50% of control and that this reduction does not alter β-1,4-xylan content of the CW.

### RGP Gene Expression

**FIGURE 4:**
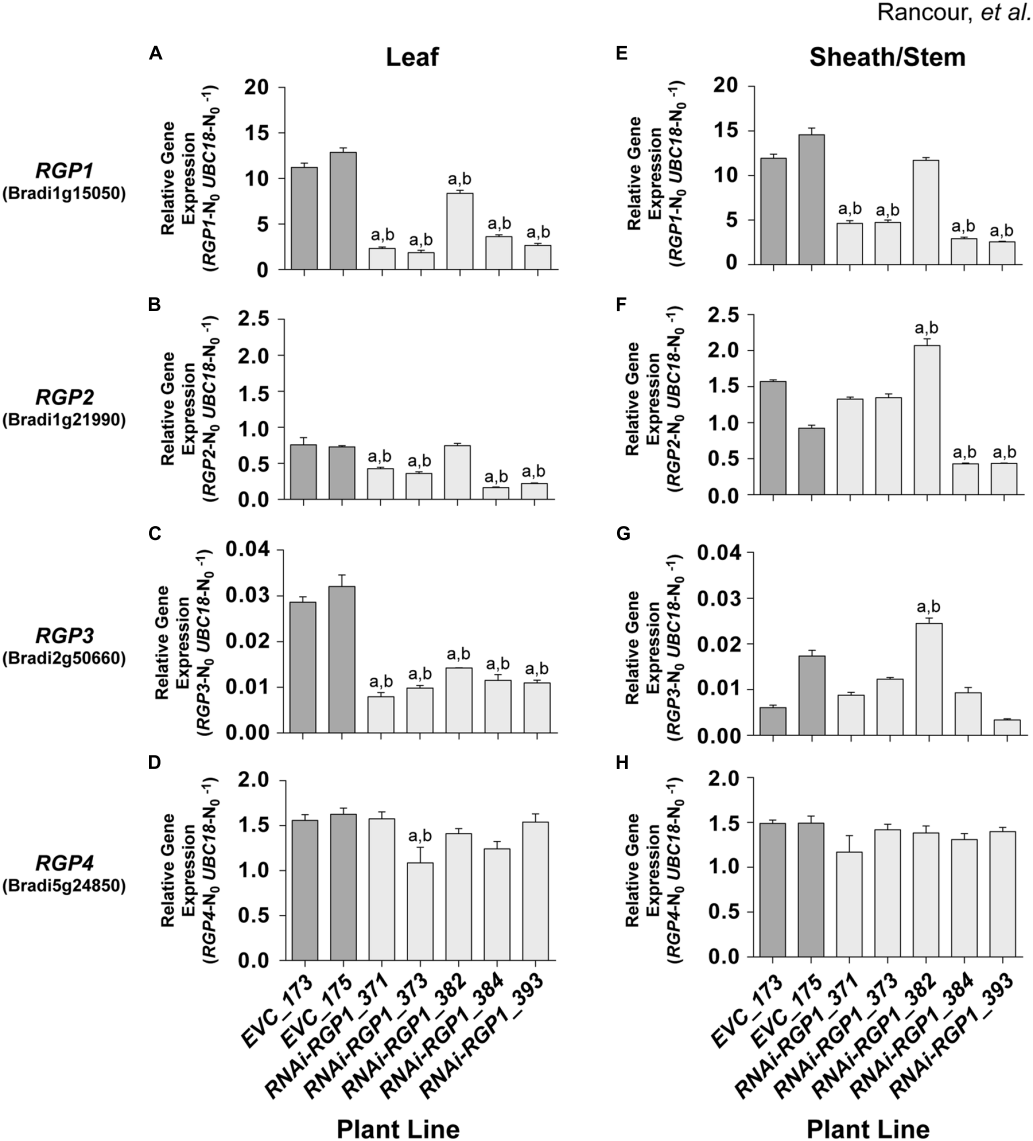
***RGP* gene-family expression in RNAi and control lines.** Real-time PCR analysis of *RGP1* **(A,E)**, *RGP2* **(B,F)**, *RGP3* **(C,G)**, and *RGP4* **(D,H)** gene expression in transgenic *B. distachyon* Bd21-3 leaf blades **(A–D)** and leaf sheath/stem **(E–H)** fractions. Empty-vector control plant lines (*EVC*) *173* and *175*, and *RNAi-RGP1* lines *371*, *373*, *382*, *384*, and *393* data are presented. Error bars indicate SEM. ^a,b^ significantly different from *EVC_173* and *EVC_175* values, respectively (ANOVA with *post hoc* Tukey test, alpha = 0.01).

Hairpin RNAi methods have been extensively used as a tool to suppress gene expression and function *in vivo* (Watson et al., 2005). To determine the efficacy of our RNAi-RGP1 construct on the *Brachypodium RGP* gene family expression, we used relative real-time PCR to assess changes in gene expression (**Figure 4**). The relative gene-specific expression of *RGP1* (Bradi1g15050; A and E), *RGP2* Bradi1g21990; B and F), *RGP3* (Bradi2g50660; C and G), and *RGP4* (Bradi5g24850; D and H) was assessed from leaf (**Figures 4A–D**) or sheath/stem (**Figures 4E–H**). We used the *BdUBC18* (Bradi4g00660) gene, which encodes ubiquitin-conjugating enzyme 18 (Hong et al., 2008), as a reference marker for real-time PCR. The uniformity of expression of *BdUBC18* across organs and development was supported in our RNA-seq data (**Figure 1A**).

*RNAi-RPG1* lines *371, 373, 384,* and *393* demonstrated significant reductions in *RGP1* gene expression throughout the organs analyzed (**Figures 4A,E**). The data indicate the abundance of target gene *RGP1* transcript in *RNAi-RGP1* lines was reduced in leaves of line *373* to 17% and in sheaths/stems in line *393* to 22% (relative to control line *EVC_173*). In line *382*, *RGP1* gene-expression was modestly reduced in leaves with no reduction detected in sheaths/stems. These data are consistent with CW analysis (**Figures 3A,B**) where lines *371, 373, 384,* and *393* all demonstrated reductions in Ara abundance, whereas line *382* did not. As expected these data indicate a strong correlation between *RGP1* gene expression and CW Ara abundance with Pearson correlation coefficients of 0.83 and 0.87 for leaves and sheath/stem samples, respectively.

However, based on the design of our RNAi-RGP1 construct through the use of a large cDNA fragment, we were also interested in whether silencing of other RGP gene family members was occurring in our *RNAi-RGP1* plants. Predictions of possible off-site targets for our RNAi construct were made using the psRNATarget online tool^3^ (Dai and Zhao, 2011) with default search parameters against the *B. distachyon* transcripts, Phytozome v8.0 option. The results (see Supplementary Table S8) suggested that other *RGP* gene family members could be targets for our RNAi-RGP1 construct as well. Therefore, using relative real-time PCR, the expression of *RGP2*, *RGP3*, and *RGP4* was determined (**Figures 4B–D,F–H**). The real-time PCR results corroborated the RNA-seq data where expression of *RGP2*, *RGP3*, and *RGP4* was at least an order of magnitude less than *RGP1*. The real-time PCR results highlighted that *RGP2* and *RGP3* expressions also were attenuated consistently in leaf tissue with lines *371*, *373*, *384,* and *393*. There was greater variability in the expression of *RGP2* and *RGP3* in sheaths/stems of control lines and *RNAi-RGP1* lines. With *RGP2* expression in sheaths/stems, control lines had nearly a 30% differential in expression.

Large variation in control-line *RGP2* expression in sheaths/stems, *RNAi-RGP1* lines *384* and *393* exhibited reductions of near 50% of the *EVC_175* value while lines *371* and *373* did not deviate from control expression levels. With *RGP3* expression in sheaths/stems, *RNAi-RGP1* lines *371*, *373*, *384,* and *393* did not change from control plant expression levels. Interestingly, expression of *RGP2* and *RGP3* increased in *RNAi-RGP1* line *382*, the line that did not maintain the reduced CW Ara phenotype observed in the T_0_ stage, but the biological significance is not known. With the expression of *RGP4*, only one line, *RNAi-RGP1_373*, had a change in expression with a 25% reduction in expression in leaf blades. The remaining lines, irrespective of tissue source, did not deviate from control plant expression levels thus calling into question the biological significance of the reduction observed in line *373*.

Considering all of the *RGP* gene family expression data together, our results indicate that the RNAi-RGP1 construct predominantly influenced expression of *RGP1, RGP2,* and *RGP3* but not *RGP4*. Expression of *RGP1, RGP2,* and *RGP3* were greatly reduced in leaves from lines *371*, *373*, *384,* and *393*. In sheaths and stems, our RNAi-RGP1 construct most affected *RGP1* expression with a varying affect on *RGP2* expression as is evident in lines *371* and *373* versus *384* and *393*. When considering gene expression with CW Ara content, it can be argued that *RGP1* and *RGP2* expression attenuation had the greatest impact on reducing CW Ara levels in our RNAi mutant *Brachypodium* plants.

### Cell Wall Hydroxycinnamates and Crosslinks are Reduced in *RNAi-RGP1* Mutant Plants

**FIGURE 5:**
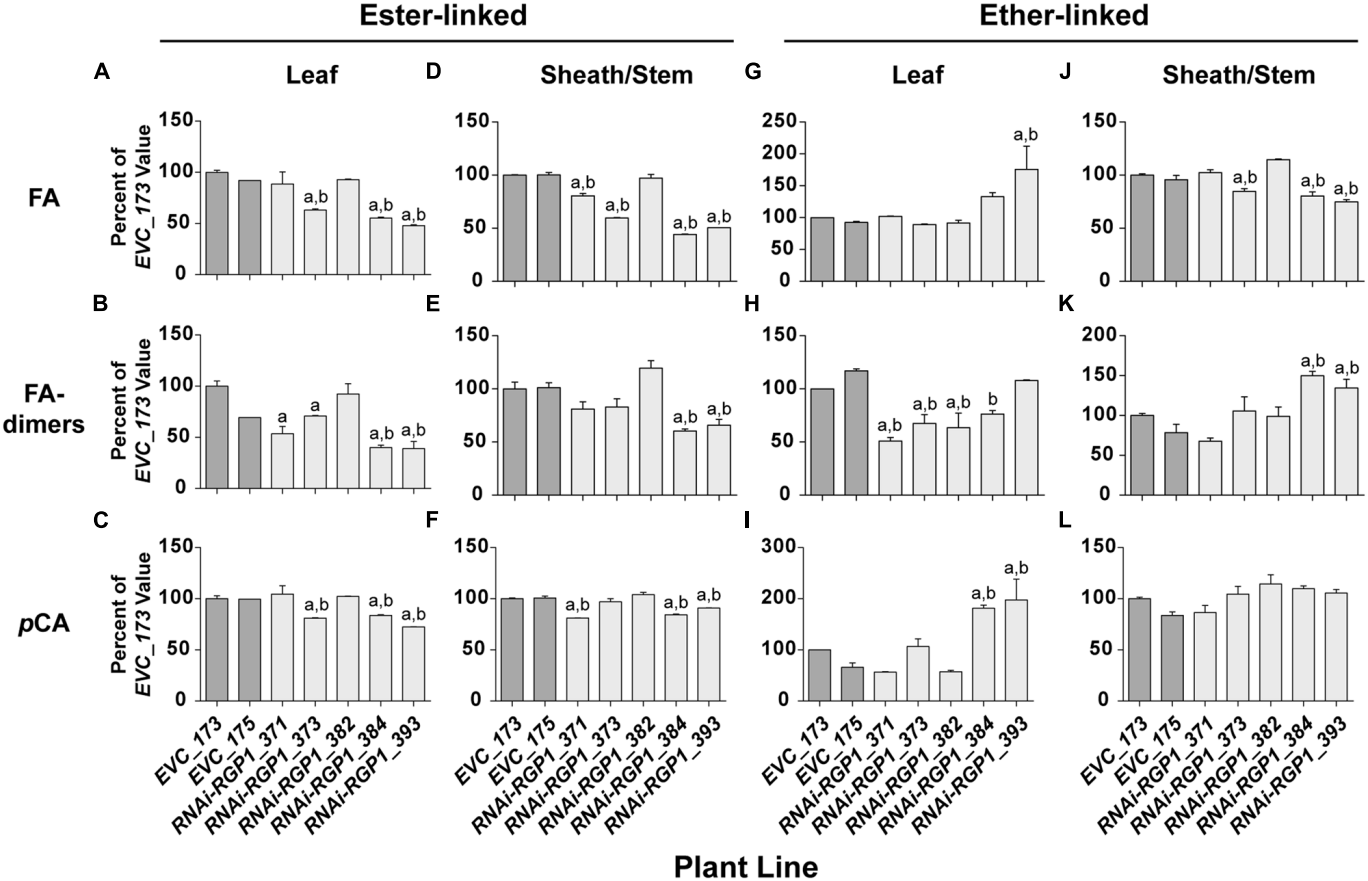
**Alterations in CW hydroxycinnamates.** CW FA **(A,D,G,J)**, FA-dimers **(B,E,H,K)** and *p*CA **(C,F,I,L)** were analyzed from leaf blade **(A–C,G–I)** or leaf sheath/stem **(D–F,J–L)** tissues of T_1_ transgenic *Brachypodium* empty-vector control lines (*173* and *175*) and *RNAi-RGP1* lines (*371*, *373*, *382*, *384*, and *393*). The hydroxycinnamate chemical linkages were distinguished as ester-linked **(A–F)** or ether-linked **(G–L)**. All values were normalized with *EVC_173* set to 100%. The *EVC_173* values (μg hydroxycinnamate mg^-1^ CW ± SEM) were 6.59 ± 0.13, 1.23 ± 0.11, 3.49 ± 0.14, 6.90 ± 0.04, 0.83 ± 0.07, 6.49 ± 0.09, 1.43 ± 0.05, 1.13 ± 0.04, 0.85 ± 0.18, 4.76 ± 0.10, 1.32 ± 0.04, and 1.43 ± 0.04, respectively for **(A–L)**. Error bars indicate SEM. ^a,b^ significantly different from *EVC_173* and *EVC_175* values, respectively (ANOVA with *post hoc* Tukey test, alpha = 0.05).

The Ara*f* modification of grass xylans provides a site for incorporation of ester-linked hydroxycinnamates into the CW. FA is esterified to the 5^′^ hydroxyl of Ara*f* and can be involved in (1) FA-dimerization forming inter-xylan crosslinks and (2) providing sites for coupling to lignin polymers (Grabber et al., 1995; Hatfield et al., 1999b; de O Buanafina, 2009). FA cross-linking has been shown to negatively correlate with CW digestibility (Grabber et al., 1998a,b). We sought to determine if reductions in CW Ara resulted in parallel reductions in FA and FA-dimers (**Figure 5**). As part of our normal analysis platform, *p*CA were also analyzed. Traditionally, *p*CA is thought to be primarily a component of lignin (Grabber, 2005; Hatfield and Marita, 2010).

Cell wall ester-linked FA and FA-dimers were reduced in *RNAi-RGP1* mutant lines when compared to controls lines (**Figures 5A,B,D,E**). Reductions were observed in both leaf and sheath/stem tissues of lines that exhibited reductions in CW Ara. The greatest reduction in FA and FA-dimer values were from lines *384* and *393* that also had the greatest reduction in CW Ara. CW ester-linked FA was reduced to as low as 48% of control values in line *393* leaves and 44% of control in sheath/stem of line *384*. Likewise, ester-linked FA-dimers were reduced to as low as 39% of control values in line *393* leaves and 60% of control in sheaths/stems of line *384*. Statistically, these data indicate that ester-linked CW FA and FA-dimers are significantly reduced in *RNAi-RGP1* mutant plants.

The abundance of ester-linked CW *p*CA also decreased in both leaves and sheaths/stems tissues of *RNAi-RGP1* mutant plants (**Figures 5C,F**). The relative decreases in *p*CA were less than those measured for FA and FA-dimers. Ester-linked *p*CA was reduced to as low as 72% of control values in line *393* leaves and 84% of control in sheath/stem of line *384*.

Ether-linked hydroxycinnamates are a result of the presence of CW hydroxycinnamates during the oxidative-coupling processes during lignification (Hatfield et al., 1999b; Ralph et al., 2004). Tissue-specific differences were observed on the release of ether-linked monomeric hydroxycinnamates (FA and *p*CA) compared to FA-dimers (**Figures 5G–L**). In leaves from strong mutant lines *384* and *393*, ether-linked monomeric hydroxycinnamate amounts increased relative to control lines but FA-dimers did not show significant change. On the contrary, leaf ether-linked FA-dimers were reduced in the weak RNAi lines *371* and *373*. In sheath/stem CWs, ether-linked FA were similar to the ester-linked FA in that strong *RNAi-RGP1* lines had the greatest reductions in FA specifically with line *393* down to 75% of control. Ether-linked *p*CA values did not change. Ether-linked FA-dimers increased in strong *RNAi-RGP1* mutant lines *384* and *393* to values of 150 and 135% of EVC_173 control value, respectively.

**FIGURE 6:**
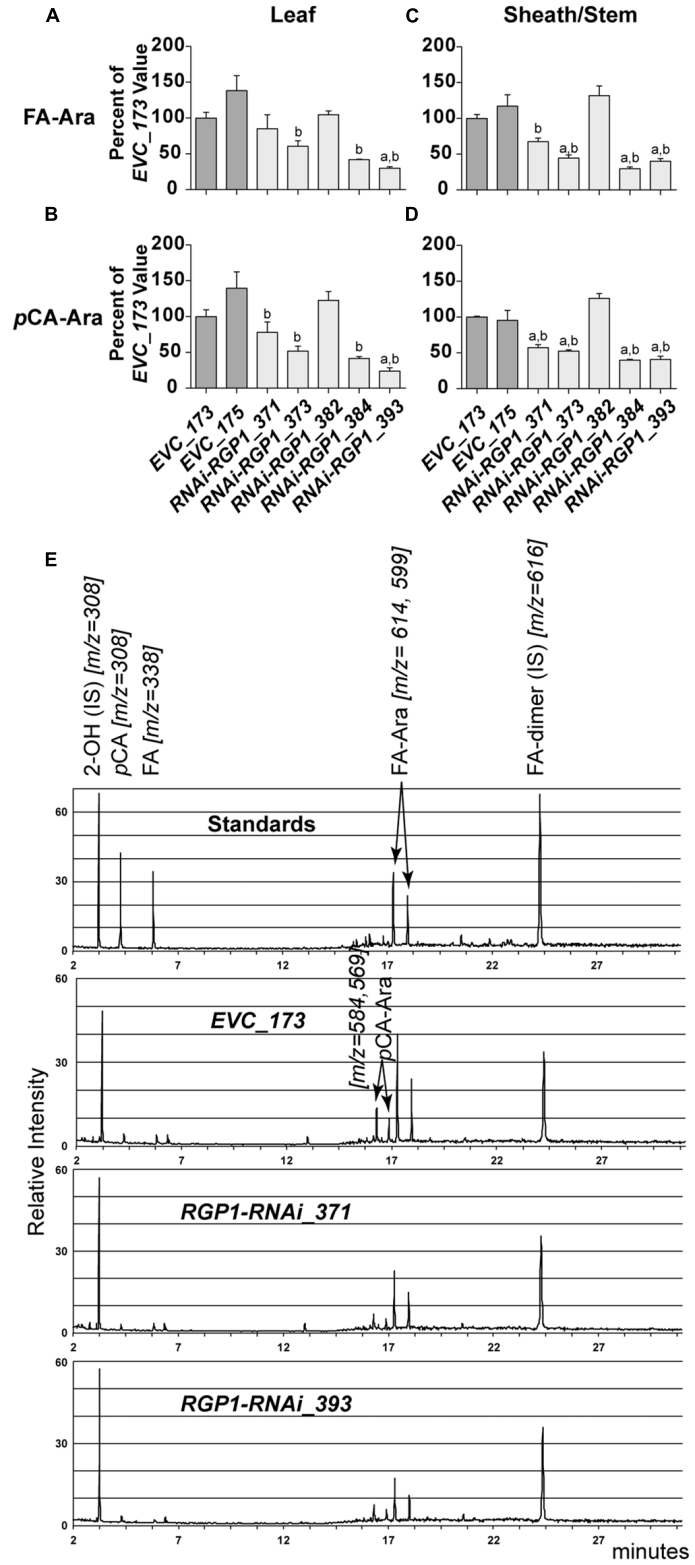
**Hydroxycinnamate-Ara conjugate analysis.** CW FA-Ara and *p*CA-Ara conjugates released from leaf blades **(A,B)** and leaf sheath/stem **(C–E)** tissues of T_1_ transgenic *Brachypodium* empty-vector control lines (*173* and *175*) and *RNAi-RGP1* lines (*371*, *373*, *382*, *384*, and *393*) were analyzed by GLC-MS. All values were normalized with *EVC_173* set to 100%. The *EVC_173* values (μg hydroxycinnamate-Ara conjugate mg^-1^ CW ± SEM) were 7.66 ± 0.43, 2.70 ± 0.18, 15.18 ± 0.59, and 5.35 ± 0.05, respectively, for **(A–D)**. Error bars indicate SEM. ^a,b^significantly different from *EVC_173* and *EVC_175* values, respectively (ANOVA with *post hoc* Tukey test, alpha = 0.05). **(E)** Stack of representative total-ion chromatograms for chemical standards, *EVC_173* stem hydrolysate, *RNAi-RGP1_371* stem hydrolysate, and *RNAi-RGP1_393* stem hydrolysate (top to bottom, respectively). Compound name and *m/z* are indicated. IS, internal standard.

The results from analysis of CW hydroxycinnamates indicate that levels of ester-linked FA, ester-linked FA-dimers, and ester-linked *p*CA decrease in strong *RNAi-RGP1* mutant lines. Current models indicated that FA and FA-dimers are directly coupled to arabinoxylans, however, *p*CA is predominantly a component of lignin. Recent work has shown that some of the CW ester-linked *p*CA is actually covalently bound to Ara of arabinoxylans in *Brachypodium* (Petrik et al., 2014). Therefore we developed and used a modified procedure for the analysis of ester-linked FA-Ara and *p*CA-Ara conjugates (**Figure 6**). The procedure uses a mild TFA hydrolysis procedure to release the FA-Ara and *p*CA-Ara moieties and the total hydrolysate is analyzed by GLC-MS with total ion chromatogram outputs (**Figure 5E**).

Analysis of CW FA- and *p*CA-Ara indicated that *RNAi-RGP1* lines that showed reductions in CW Ara levels exhibited reductions in both FA-Ara (**Figures 6A,C**) and *p*CA-Ara (**Figures 6B,D**) compared to *EVC*-lines. The trends of decreased FA-Ara and *p*CA-Ara followed relative decreases in ester-linked FA, *p*CA, and CW Ara abundances. It is expected that FA-Ara reductions would be comparable to the total ester-linked FA reduction assuming that FA-Ara is the sole source of CW ester-linked FA. However, the proportional reductions in *p*CA-Ara are much greater than the reductions in total ester-linked *p*CA. For example *p*CA-Ara was reduced by as much as 60% in *RNAi-RGP1_384* leaves and 76% in *RNAi-RGP1_393* sheaths/stems while the reductions in total ester-linked *p*CA was only 16 and 28%, respectively. Comparing our data, we determined that *p*CA-Ara conjugates comprise approximately 50% of the total ester-linked *p*CA in *B. distachyon* and that the reduction in ester-linked *p*CA observed in strong *RNAi-RGP1* lines is likely derived from *p*CA-Ara.

The molar ratio of CW monomeric FA (ester- and ether-linked) to Ara can indicate changes in the modification frequency of Ara by FA. Our data showed only one statistically significant change in the normalized organ-specific CW FA to Ara ratio between *RNAi-RGP1* mutants and *EVC*-lines (Supplementary Figure S8). The only statistically significant difference occurs when comparing sheath/stem CW FA to Ara of *EVC_173* to *RNAi-RGP1_371* (Supplementary Figure S8B). All remaining comparisons were not statistically significant. Taken in their entirety, the data suggest that the FA substitution frequency on Ara is not directly impacted by the RGPs.

With changes in CW hydroxycinnamates and Ara detected in *RNAi-RGP1* lines, we sought to determine if changes occurred in total lignin and lignin composition. Total CW lignin was determined by the acetyl bromide lignin method as described (Fukushima and Hatfield, 2001; Rancour et al., 2012). The lignin concentration was significantly changed only in leaf CWs from *RNAi-RGP1_384* and 393 lines where total lignin was increased by 29 and 21%, respectively (Supplementary Figure S9A). The results indicated that no significant differences in total lignin were present between *EVC* and *RNAi-RGP1* lines in sheath/stem tissue (Supplementary Figure S9B).

To determine if lignin composition changes occurred, gel-state 2D NMR analysis of total CWs isolated from WT_21-3 and *RNAi-RGP1_373* and 393 lines was performed as described (Kim and Ralph, 2010; Marita et al., 2014) to quantify lignin subunit ratios (syringyl to guaiacyl) and the relative syringyl to *p*CA composition. Reliable quantification of the aromatic unit ratios is dependent on the integrations of well-dispersed 2- and/or 2,6- positions of each aromatic unit. The 2D HSQC experiment performed on the *Brachypodium* CWs accounts for all of a given phenolic component (all guaiacyl and syringyl units) within the CW. The syringyl to guaiacyl subunit ratios differed modestly in leaves with values of 0.44 for WT_21, 0.48 for *RNAi-RGP1_373*, and 0.68 for *RNAi-RGP1_393*. In addition to changes in the syringyl:guaiacyl (S:G) lignin subunit ratio, a modest change in the leaf syringyl:*p*CA ratio was observed with values of 1.06 for WT_21, 1.66 for *RNAi-RGP1_373*, and 2.42 for *RNAi-RGP1_393*. Only among lines with the greatest reduction in *RGP* expression do we detect changes in total lignin and lignin composition. These lignin alterations appear confined to leaf blade walls but the biological significance is not known. Taken together, reductions in CW hydroxycinnamates and Ara do not correlate with drastic whole-plant alterations in total lignin and lignin composition.

### Digestibility of *RNAi-RGP1* CWs

**FIGURE 7:**
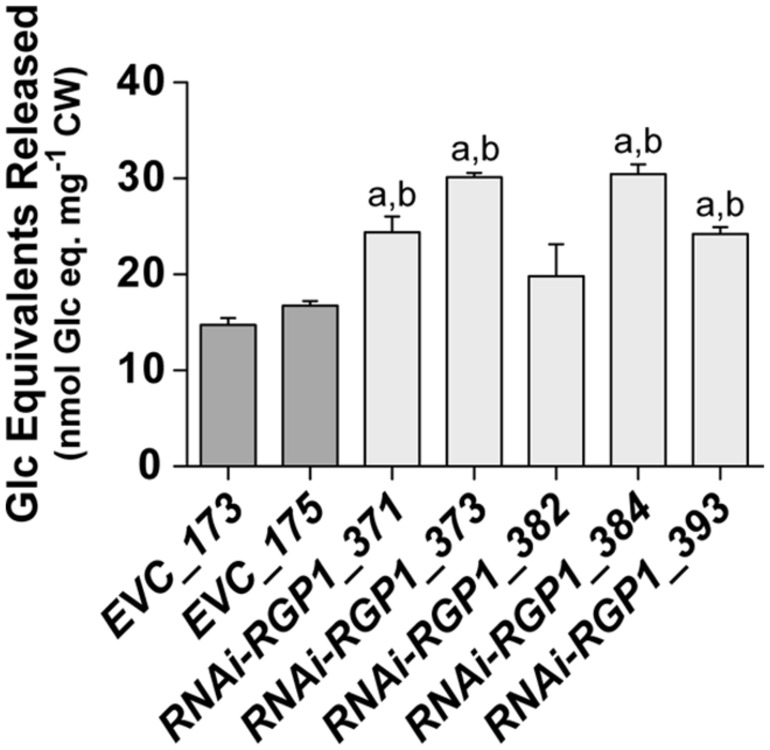
**Increased CW digestibility of Ara-deficient mutant *B. distachyon* with xylanase.** Total sugar released from *Thernomyces lanuginosus* endo-1,4-β-xylanase-treated sheath/stem CWs of T_1_ transgenic *Brachypodium* empty-vector control lines (*173* and *175*) and *RNAi-RGP1* lines (*371*, *373*, *382*, *384*, and *393*) is presented. Error bars indicate SEM. ^a,b^ significantly different from *EVC_173* and *EVC_175* values, respectively (ANOVA with *post hoc* Tukey test, alpha = 0.05).

The digestibility of plant biomass is an important feature in forage utilization and bioenergy applications. Numerous factors, including lignin and FA-crosslinking, have been proposed to negatively impact digestibility. With the observed reductions of CW ester-linked hydroxycinnamates, including FA and FA-dimers, in our *RNAi-RGP1* plants, we sought to determine if these plants had alterations in the enzyme-mediated digestibility characteristics. Firstly, sheath/stem CWs were treated with *T. lanuginosus* endo-1,4-β-xylanase overnight and total sugar release was measured (**Figure 7**). Without any pre-treatment of the CWs, we observed increases in released CW carbohydrate of up to twofold from *RNAi-RGP1* lines compared to *EVC*-lines. Carbohydrate released from *EVC*-lines was 14.7-16.7 nmol Glc equivalents per mg CW. The *RNAi-RGP1* lines with notable Ara and hydroxycinnamate reductions exhibited carbohydrate release amounts of 24.2–30.5 nmol Glc equivalents per mg CW. These data indicate that reductions of CW-bound Ara and hydoxycinnamates in grasses without changing lignin levels can lead to improved xylan digestibility.

**FIGURE 8:**
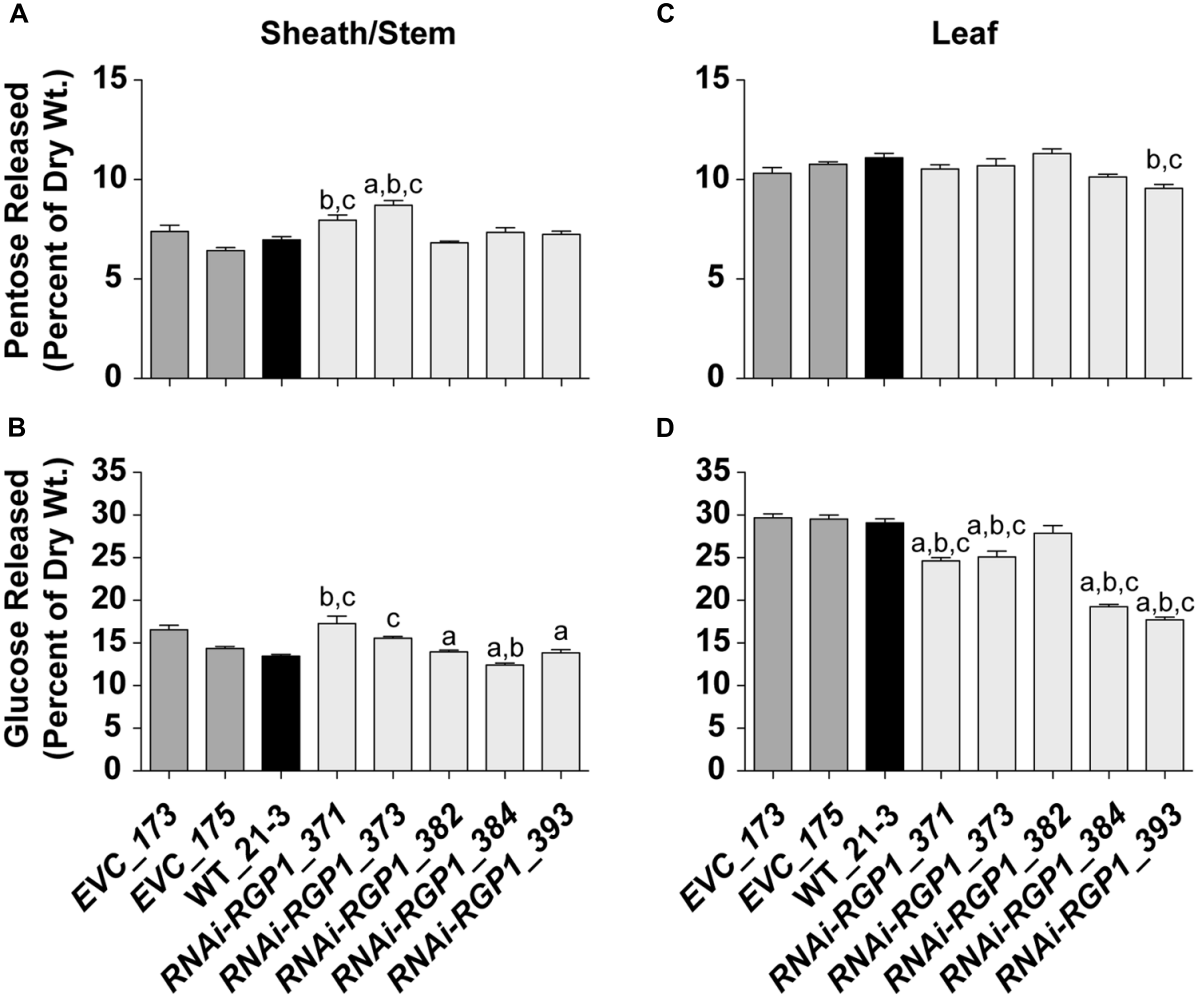
**Saccharification of Ara-deficient mutant *B. distachyon* CWs with Accelerase 1000 enzyme mix.** Pentose **(A,C)** and Glc **(B,D)** released from Accelerase 1000-treated sheath/stem **(A,B)** and leaf **(C,D)** CWs of T_1_ transgenic *Brachypodium empty-vector* control lines (*173* and *175*), wild-type (WT_21-3), and *RNAi-RGP1* lines (*371*, *373*, *382*, *384*, and *393*) is presented. Error bars indicate SEM. ^a,b,c^ significantly different from *EVC_173*, *EVC_175*, and WT_21-3 values, respectively (ANOVA with *post hoc* Tukey test, alpha = 0.05).

To determine if total CW digestibility was improved with *Brachypodium RGP* suppression, saccharification was assessed using the Accelerase 1000 enzyme mixture (Santoro et al., 2010) on sheath/stem and leaf CWs based on Santoro et al. (2010) (**Figure 8**). CW material was pretreated with 6.25 mM NaOH. Release of CW Glc and pentoses were measured and those data are presented. Pentose release from mutant sheath/stem CWs was improved in the weak expressing *RNAi-RGP1* mutants *371* and *373* (**Figure 8B**). *RNAi-RGP1* line *373* exhibits an increase of 18% over *EVC_173* control. With the stronger expressing *RNAi-RGP1* mutant alleles, *384* and *393*, apparent sheath stem pentose release was unaffected. Pentose release from leaf CWs was mostly unaffected except for a decrease observed in the strong expressing *RNAi-RGP1* line *393* (**Figure 8C**).

Glucose release from *RNAi-RGP1* CWs exhibited mixed and unexpected results (**Figure 8B,D**). Sheath/stem Glc release was not consistently altered across *RNAi-RGP1* lines when compared to control lines (**Figure 8B**). Weak expressing *RNAi-RGP1* lines trended with increased Glc release while strong expressing lines showed decreases in Glc release from sheath/stem CWs. Surprisingly, Glc release was reduced significantly from leaf CWs of all *RNAi-RGP1* lines (**Figure 8D**). The reduction in leaf Glc release positively correlated with CW Ara content and the relative reductions in *RGP1* and *RGP2* gene expression with Pearson correlation coefficients of 0.90, 0.78, and 0.94, respectively. These data indicate organ-specific effects on CW composition translate to differing consequences on CW digestibility depending on the enzyme treatment being applied.

## Discussion

The acknowledgment of climate change and the need to support the growing global human population’s need for food has put high demand on technology development to maximize plant biomass production and utilization for sustainable and renewable sources of food, feed, fiber, and energy (FAO, 2003). To move forward with research to address these needs requires a clear understanding of how processes occur from gene expression to final assembly in a functioning plant. Approaches to improve biomass quality and use-efficiency while not grossly impacting yields are needed.

The plant CW is a complex mixture of carbohydrates, aromatic-compounds, and protein that is critical for plant form and function but is yet poorly understood in how it is assembled and managed throughout plant development. The content of the plant CW suggests it is a metabolic sink for cell carbohydrate. It is this carbohydrate that is most useful in terms of feed, fiber, and bioenergy and its utilization is dependent upon overcoming the limitations in its efficient extraction from plants (CWs).

Opposing but not mutually exclusive views can be taken to improve carbohydrate removal including (1) making the material more “extractable” and (2) increasing the yield of “target” per unit CW. Much work addressing the former has focused on decreasing or altering inhibitors to extractability such as lignin (Vanholme et al., 2012). Alternatively, proposals on increasing cellulose (Delmer and Haigler, 2002) yield have been made. However, approaches focused on cellulose synthase have been hampered due to the complexity of the gene and enzyme regulation (Somerville, 2006; Mutwil et al., 2008; Tan et al., 2015). Recent work suggests that targeted increases in β-glucans ((1,3;1,4)-β-D-glucans) might prove feasible (Burton et al., 2011). The recent identification and manipulation of regulatory transcription factors has led to ectopic initiation of secondary CW biosynthesis (Wang and Dixon, 2012; Valdivia et al., 2013). However, the targets of these studies were not specific enough to cause beneficial changes in carbohydrate yields without increasing the inhibitory CW aromatics. Recent work in Arabidopsis provides an example of new methods using cell-type specific promoter swapping and engineered feedback loops that can be used to decrease lignin and increase CW carbohydrates (Yang et al., 2013). The translation of these studies to grasses has not been reported.

In grasses, the CW carbohydrate composition is dominated by Glc, Xyl, and Ara (Pauly and Keegstra, 2008). Given that the CW is a metabolic sink for carbohydrate, we hypothesized that manipulation of the biosynthesis of the nucleotide sugar substrates for CW polysaccharide biosynthesis could lead to beneficial changes in CW composition. Our approach was to systematically target steps in the linear metabolic pathway for conversion of UDP-α-D-Glc to UDP-β-L-Ara*f* in *B. distachyon* through either RNAi-mediated gene suppression or constitutive gene expression of genes encoding enzymes within this pathway. We envisioned several potential outcomes. One example, suppression of the UGD, the first committed step in the pathway, may lead to a metabolic back-up of cytoplasmic UDP-Glc (as seen in Behmüller et al., 2014) that could lead to greater substrate availability for cellulose synthase and/or β-glucan synthesis and thus potentially stimulate their enzyme activity while relying on endogenous gene expression. In another example, overexpression of a UGD or a UXS could lead to increased xylan production (as seen in alfalfa, Samac et al., 2004). Finally, reductions in the ability to synthesize Ara by suppression of either the UXE or the UDP-Ara*p* mutase may lead to alterations in arabinoxylan abundance and structure thus influencing total FA-mediated crosslinking (Hatfield et al., 1999b; de O Buanafina, 2009) and potentially improving digestibility.

Therefore, we took an approach to generate transgenic *B. distachyon* plants and select them based on two criteria (1) altered CW carbohydrate profiles and (2) near normal overall stature of the plant. From an agronomic perspective, the latter criterion is important to ensure that possible improvements in digestibility are not negated by decreases in yields as observed previously (Reddy et al., 2005; Chen and Dixon, 2007; Shadle et al., 2007). Plants derived from transformation with the RNAi-RGP1 (UDP-Ara*p* mutase-1) construct met these criteria and were further characterized.

Analysis of multiple T_1_ *RNAi-RGP1* lines shows that simultaneous suppression of *RGP1* and *RGP2* in *Brachypodium* results in decreased CW-bound Ara and hydroxycinnamates. Our data suggests that these CW reductions can result in modest improvements in xylan digestibility but surprisingly, little improvement in cellulose and/or β-glucan digestibility as indicated by Glc release from the CW (**Figures 8B,D**). In addition, our results suggest organ-specific responses to the reduction in RGP expression. In leaf blades, though reductions on CW-bound Ara and hydroxycinnamates were comparable to sheath/stem values, the responses of increased lignin in strong expressing *RNAi-RGP1* lines (Supplementary Figure S9) as well as the marked reduction of Glc release in all *RNAi-RGP1* lines indicate the CW alterations have differing organ-specific affects. The decreased cellulose digestibility may be due to a combination of improved hydrogen bonding of undecorated xylan backbones with cellulose fibrils, organ-specific expression of CW protein(s), and/or increased lignification in response to altered arabinoxylan structure/function. Xylan *sans* Ara would be linear and suited for strong hydrogen bonding interactions with cellulose and decreased binding accessibility for hydrolytic enzymes. These interactions may be compounded through organ-specific CW protein expression. Additionally, it is unclear how alterations in arabinoxylan structure are sensed and translated into responses of increased lignification. Further experiments are needed to test these hypotheses. These current experiments provide proof-in-principle that UDP-Ara*f* biosynthesis could be a target in grasses for designing crops with altered biomass composition without compromising yield. Earlier work by Jung et al. (2011) indicated that reduced ferulates and ferulate mediated cross-linking resulted in increased digestibility of corn silage by dairy cows. Although the available amounts of *RNAi-RGP1* material in this study was not sufficient to test digestibility in an animal system, it is most likely that the reduced levels of ferulate crosslinking would result in increased CW digestibility. Microbial ecosystems such as those found in ruminants can do a much better job of breaking down complex CWs compared to individual enzymes. The large array of different microbial species within the rumen can quickly take advantage of niche openings cause by relatively small changes in the CW matrix, e.g., decreased ferulate cross-linking.

Although the *RNAi-RGP1* lines did meet the goal of altered CW composition without major disruption in plant stature, the question remains as to why other constructs did not result in CW compositional changes? Some possibilities for this are presented. (1) The majority of constructs were ineffective in achieving sufficient gene suppression or constitutive expression to influence overall metabolism that manifests as CW composition phenotypes. This could be due to choosing the incorrect gene involved in CW substrate biosynthesis. Alternatively, it is not known how much catalytic flux capacity exists in excess of CW requirements and thus how much down-regulation can occur before effects in CW synthesis are observed. Little is known about nucleotide sugar metabolic flux in grasses and how it feeds into the regulation of CW synthesis and other utilizations such as glycoprotein and glycolipid biosynthesis. Analytical platforms have been developed recently to begin addressing the size of plant nucleotide–sugar pools and the flux through them (Alonso et al., 2010; Pabst et al., 2010; Chen et al., 2013a; Behmüller et al., 2014; Ito et al., 2014; Rautengarten et al., 2014). Using new HPLC-MS methods, steady state concentrations of UDP–sugars have been quantified from Arabidopsis T87 cultured cells (Alonso et al., 2010) and leaves (Pabst et al., 2010; Behmüller et al., 2014; Ito et al., 2014), tobacco leaves (Pabst et al., 2010), and rice leaves (Ito et al., 2014). A recent study by Rautengarten et al. (2014) reported on steady-state pools of 13 nucleotide–sugars in nine different *Arabidopsis* organs and developmental stages. In support of our approach, the work of Behmüller et al. (2014) demonstrated that steady state levels of UDP–sugars downstream of UDP-Glc were reduced in leaves from *Arabidopsis ugd2,3* double-mutant plants but the work did not look at whether CW composition phenotypes resulted. Recent metabolic control analysis using *Arabidopsis* T87 suspension-cultured cells highlighted possible metabolic points where alterations in flux would have pronounced effects on the incorporation of Xyl into a dicot CW (Chen et al., 2013a). It would be of great interest to extend this work to monocots due to their differing CW composition and the number of monocot species used for forage and lignocellulosic biofuel production.

(2) The procedure for *Brachypodium* transformation involves generation of transgenic embryonic callus followed by plant regeneration. This regeneration process is complicated and presumably involves distinct requirements of the CW in meeting mechanical and compositional needs to ensure multicellular development progresses appropriately. Given this complexity, manipulation of select metabolic genes to extreme levels (a) could inhibit plant regeneration via CW, glycoprotein, and or glycolipid defects to the point where no plants survived regeneration or (b) mutants with CW phenotypes were culled from our selection process due to not meeting our plant stature requirement. Our results indicate a tolerance in grasses for reduced Ara levels attached to arabinoxylans. UDP-GlcA and UDP-Xyl are inputs for the biosynthesis of pectins and xylans. We speculate that there might be less tolerance for manipulation of UDP-GlcA and UDP-Xyl levels in grasses. Recent work in rice showed decreases in culm CW xylan (decrease of CW Xyl by 14%) via a loss-of-function glycosyltransferase mutation is achievable resulting in a 29% increase in total sugar released upon saccharification (Chen et al., 2013b). However, it is unclear whether manipulation of xylan and/or pectins is achievable via substrate biosynthesis limitation. Assuming a strong effect on xylan biosynthesis by RNAi-mediated inhibition of UDP-Xyl synthesis, our constitutive promoter RNAi system may not be best suited to adequately test the relationship of UDP-Xyl production to xylan biosynthesis. Having a constitutive inhibition system may pose issues if xylans and/or pectins are critical for early plant development. To circumvent these possible developmental consequences, it would be interesting to explore the use of chemical-inducible systems and/or the use of tissue-/developmental-specific promoters to drive expression of constructs in manipulating nucleotide sugar metabolism in grasses for improved CW composition and digestibility phenotypes. These endeavors are for future work.

Our work has demonstrated that the RGP/UAM gene family can be viable targets for engineering plant CWs with altered CW composition. Using RNAi-mediated gene suppression, Konishi et al. (2011) demonstrated that suppression of *OsUAM1* in rice resulted in CW Ara and FA changes. However, the mutant rice plants exhibited dwarfed phenotypes, presumably based on screening for the most severe mutant plants. Their analysis did not include measurement of FA-dimers, important for CW crosslinking, or assessment of CW digestibility. Our work highlights that the selection of mutant plants with normal growth habit is an important and achievable criteria to consider in identifying plant lines with improved biomass characteristics.

## Conclusion

Through the use of RNAi-mediated gene suppression in *B. distachyon*, we have shown that *RNAi-RGP1* plants with near wild-type stature can have significant reductions in *RGP1* and *RGP2* gene expression, and exhibit reductions in CW-bound Ara and hydroxycinnamate content throughout vegetative aerial plant CWs. The sheath/stem CWs of these plants exhibit up to twofold xylanse digestibility improvements over controls. However, *in vitro* enzyme-mediated release of Glc from leaf CWs is impeded in *RNAi-RGP1* mutants. These data support the efficacy of a selection-scheme where mutant plants of near wild-type stature are screened for CW composition phenotypes. In addition, these data highlight the complexity of grass CW composition and ways to manipulate that complexity to improve digestibility.

## Author Contributions

All authors of the manuscript meet the essential requirements for publication.

## Conflict of Interest Statement

The authors declare that the research was conducted in the absence of any commercial or financial relationships that could be construed as a potential conflict of interest.

## Footnotes

1 www.roche-applied-science.com/webapp/wcs/stores/servlet/CategoryDisplay?catalogId=10001&tab=Assay+Design+Center&identifier=Universal+Probe+Library&langId=-1

2 http://www.quantprime.de/main.php?page=home

3 http://plantgrn.noble.org/psRNATarget/

## Abbreviations

Ara: arabinose
Ara*f*: arabinofuranose
Ara*p*: arabinopyranose
AXS: UDP-apiose/UDP-xylose synthase
CW: cell wall
FA: ferulic acid
Fuc: fucose
Gal: galactose
Glc: glucose
Man: mannose
*p*CA: *p*-coumaric acid
RGP: reversibly glycosylated protein
Rha: rhamnose
RNAi: RNA-mediated interference
UAM: UDP-arabinopyranose mutase
UDP-Ara*f*: uridine diphospho arabinofuranose
UDP-Ara*p*: uridine diphospho arabinopyranose
UGD: UDP-glucose dehydrogenase
UXE: UDP-xylose epimerase
UXS: UDP-xylose synthase
Xyl: xylose
