# Supplementary material for: Cell wall composition and digestibility alterations in Brachypodium distachyon achieved through reduced expression of the UDP-arabinopyranose mutase
Source: Front Plant Sci. 2015 Jun 17;6:446. doi: 10.3389/fpls.2015.00446 (PMC4470266; doi:10.3389/fpls.2015.00446)
Supplement: Supplementary file 2 [file Image_1.PDF]

Supplemental Data Legends

Supplemental Figure S1. UDP-α-D-Glc dehydrogenase (UGD) protein sequence alignment and phylogenetic tree

(A) Protein alignment of predicted UGD enzyme sequences for *Arabidopsis thaliana*, rice, *Brachypodium distachyon* Bd21, and maize. Majority consensus sequence is on top. Amino acid deviations from the majority consensus sequence are indicated for each protein sequence. (B) Phylogenetic tree for predicted UGD enzyme sequences aligned in (A).

Supplemental Figure S2. UDP-α-D-xylose synthase (UXS) protein sequence alignment

Protein alignment of predicted UXS enzyme sequences for *Arabidopsis thaliana*, rice, *Brachypodium distachyon* Bd21, and maize. Majority consensus sequence is on top. Amino acid deviations from the majority consensus sequence are indicated for each protein sequence.

Supplemental Figure S3. UDP-α-D-xylose synthase (UXS) phylogenetic tree

Phylogenetic tree for predicted UXS enzyme sequences aligned in Sup. Fig. S2.

Supplemental Figure S4. UDP-α-D-xylose epimerase (UXE) protein sequence alignment and phylogenetic tree

(A) Protein alignment of predicted UXE enzyme sequences for *Arabidopsis thaliana*, rice, *Brachypodium distachyon* Bd21, and maize. Majority consensus sequence is on top. Amino acid deviations from the majority consensus sequence are indicated for each protein sequence. (B) Phylogenetic tree for predicted UXE enzyme sequences aligned in (A).

Supplemental Figure S5. UDP-β-L-arabinopyranose mutase (RGP/UAM) protein sequence alignment and phylogenetic tree

(A) Protein alignment of predicted RGP enzyme sequences for *Arabidopsis thaliana*, rice, *Brachypodium distachyon* Bd21, and maize. Majority consensus sequence is on top. Amino acid deviations from the majority consensus sequence are indicated for each protein sequence. (B) Phylogenetic tree for predicted RGP enzyme sequences aligned in (A).

Supplemental Figure S6. UDP-α-D-apiose/UDP-α-D-Xyl synthase (AXS) protein sequence alignment and phylogenetic tree

(A) Protein alignment of predicted AXS enzyme sequences for *Arabidopsis thaliana*, rice, *Brachypodium distachyon* Bd21, and maize. Majority consensus sequence is on top. Amino acid deviations from the majority consensus sequence are indicated for each protein sequence. (B) Phylogenetic tree for predicted AXS enzyme sequences aligned in (A).

Supplemental Figure S7. Cloned cDNA sequences

FASTA formatted sequences for cDNAs used in this study. Upper case indicates putative coding sequence. Lower case text indicates UTRs (5′ or 3′) contained within clones. Black sequences were cloned and sequence verified. Blue sequences were derived from predictions based on the *Brachypodium distachyon* Bd21 genome sequence and were used for primer design and bioinformatics.

Supplemental Figure S8. FA to Ara ratios

(A) Leaf blade and (B) leaf sheath/stem normalized cell wall FA to Ara molar ratios from tissues of T_1 transgenic *Brachypodium* empty-vector control lines (*173* and *175*) and *RNAi-RGP1* lines (*371*, *373*, *382*, *384*, and *393*). All values were normalized with *EVC_173* values set to 100%. The values for *EVC_173* were 0.113±0.012 and 0.243±0.007, respectively for leaf and sheath/stems. Error bars indicate SEM. [a] significantly different from *EVC_173* value (ANOVA with *post hoc* Tukey test, alpha=0.05).

Supplemental Figure S9. Cell wall lignin

(A) Leaf blade and (B) leaf sheath/stem cell wall lignin concentrations from tissues of T_1 transgenic *Brachypodium* empty-vector control lines (*173* and *175*) and *RNAi-RGP1* lines (*371*, *373*, *382*, *384*, and *393*) were determined according to the acetyl bromide method. Error bars indicate SEM. [a,b] significantly different from *EVC_173* and *EVC_175* values, respectively (ANOVA with *post hoc* Tukey test, alpha=0.05).

Supplemental Table S1. All primers and oligonucleotides used
Total list of all primers used in this study.
Supplemental Table S2. Gene specific primers and their uses
Summary table of gene-specific primers and their uses in this study.
Supplemental Table S3. cDNA mutations versus Bd21 genome sequence: A summary
A comparison of the Bd21-3 cDNA clone sequences used in this study compared to the Bd21 genome sequence (International_Brachypodium_Initiative, 2010). Identification, location, and nature of the mutations, if any, are given.
Supplemental Table S4. cDNA mutants for RNAi vector construction
Summary table of cDNA deletion mutants used to generate RNAi constructs. Listed are the endonuclease enzyme(s) used for deletion construction, and the properties of the cDNA region retained and removed. The retained region was further used for RNAi vector construction.
Supplemental Table S5. Representative T_0 carbohydrate screen results.
T_0 cell wall carbohydrate results including total amounts (μmol g^{-1} CW) of Ara, Xyl, and Glc, and molar ratios of Ara/Xyl and Xyl/Glc. Color highlighting corresponds to whether value is at least one or two standard deviation different than wild-type (WT) control values. Color highlighting corresponds to: Red- at least 1 SD less than WT; Blue- at least 2 SDs less than WT; Yellow- at least 1 SD greater than WT; Green- at least 2 SDs greater than WT.
Supplemental Table S6. Cell wall yields
Genotype, line and tissue, fresh weight input weight (g), dry cell wall output weight (g), and the weight percentage yield of cell wall to tissue input are given. *EVC*, empty vector control.
Supplemental Table S7. T_1 cell wall neutral sugar analysis results
Neutral sugar analysis of control (WT_21-3, *EVC_173*, and *EVC_175*) and *RNAi-RGP1* lines (*371*, *373*, *382*, *384*, and *393*) lines for leaf and sheath/stem cell walls. Values represent μmol sugar g^{-1} cell wall ±SEM (n-=4). Highlighted values correspond to statistically significant differences compared to controls (ANOVA with *post hoc* Tukey test, alpha=0.05): yellow-both *EVC lines* and wild-type; blue- *EVC_173* and wild-type; green- *EVC_175*. Values not highlighted showed no statistically significant differences to any control lines.
Supplemental Table S8. Predicted off-target sites for RNAi-RGP1 construct
Output data for psRNATarget analysis (Dai and Zhao, 2011) of RNAi-RGP1 construct giving possible alternative gene targets affected by the RNAi-RGP1 construct.

Supplemental Figure S1.

A

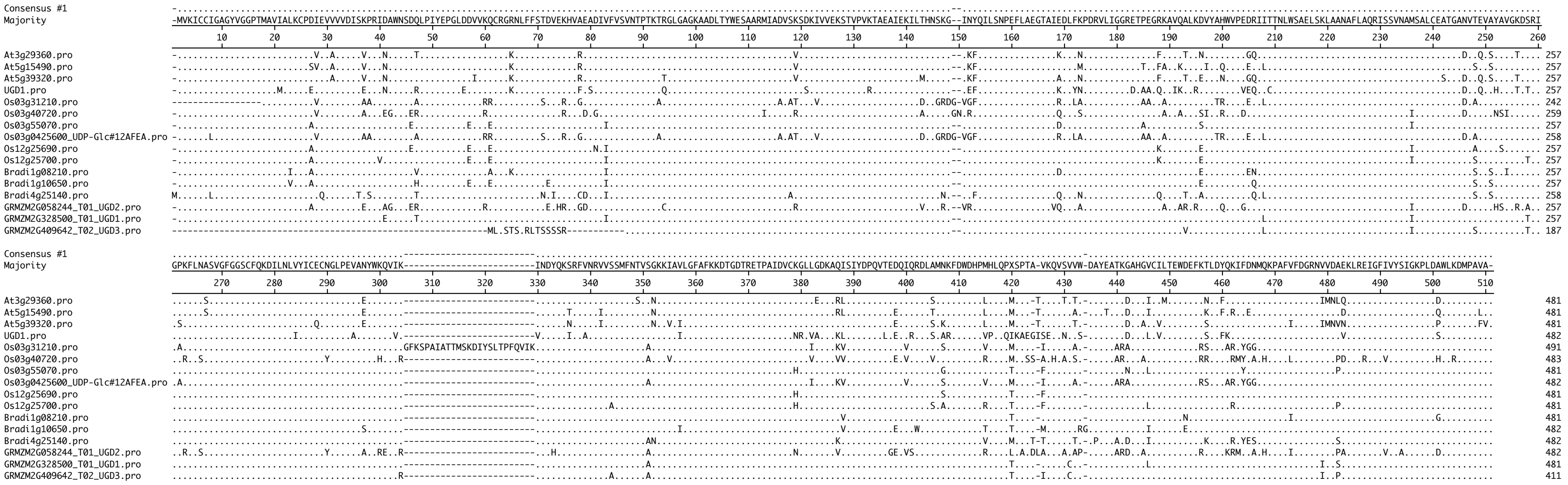

B

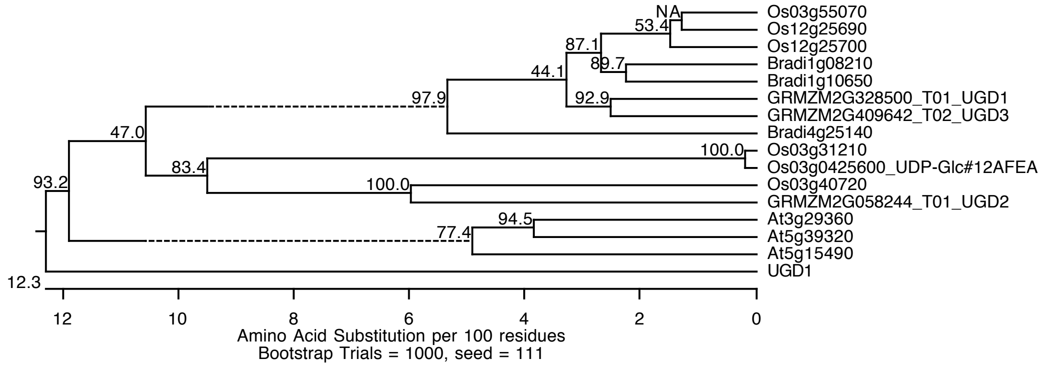

Supplemental Figure S1. UGD protein sequence alignment and phylogenetic tree

(A) Protein alignment of predicted UGD enzyme sequences for *Arabidopsis thaliana*, rice, *Brachypodium distachyon* Bd21, and maize. Majority consensus sequence is on top. Amino acid deviations from the majority consensus sequence are indicated for each protein sequence.

(B) Phylogenetic tree for predicted UGD enzyme sequences aligned in (A).

Supplemental Figure S2.

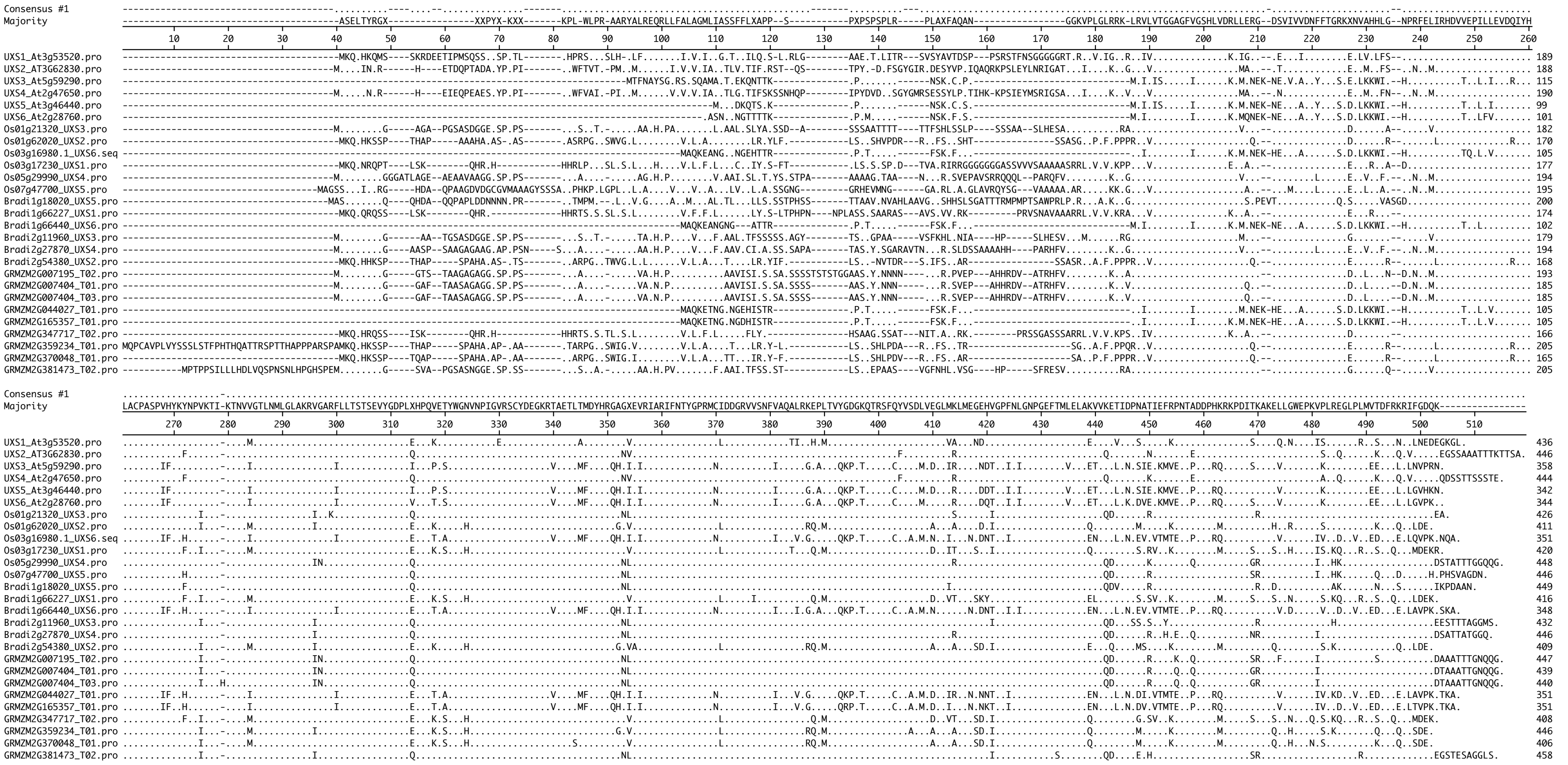

Supplemental Figure S2. UXS protein sequence alignment

Protein alignment of predicted UXS enzyme sequences for *Arabidopsis thaliana*, rice, *Brachypodium distachyon* Bd21, and maize. Majority consensus sequence is on top. Amino acid deviations from the majority consensus sequence are indicated for each protein sequence.

Supplemental Figure S3.

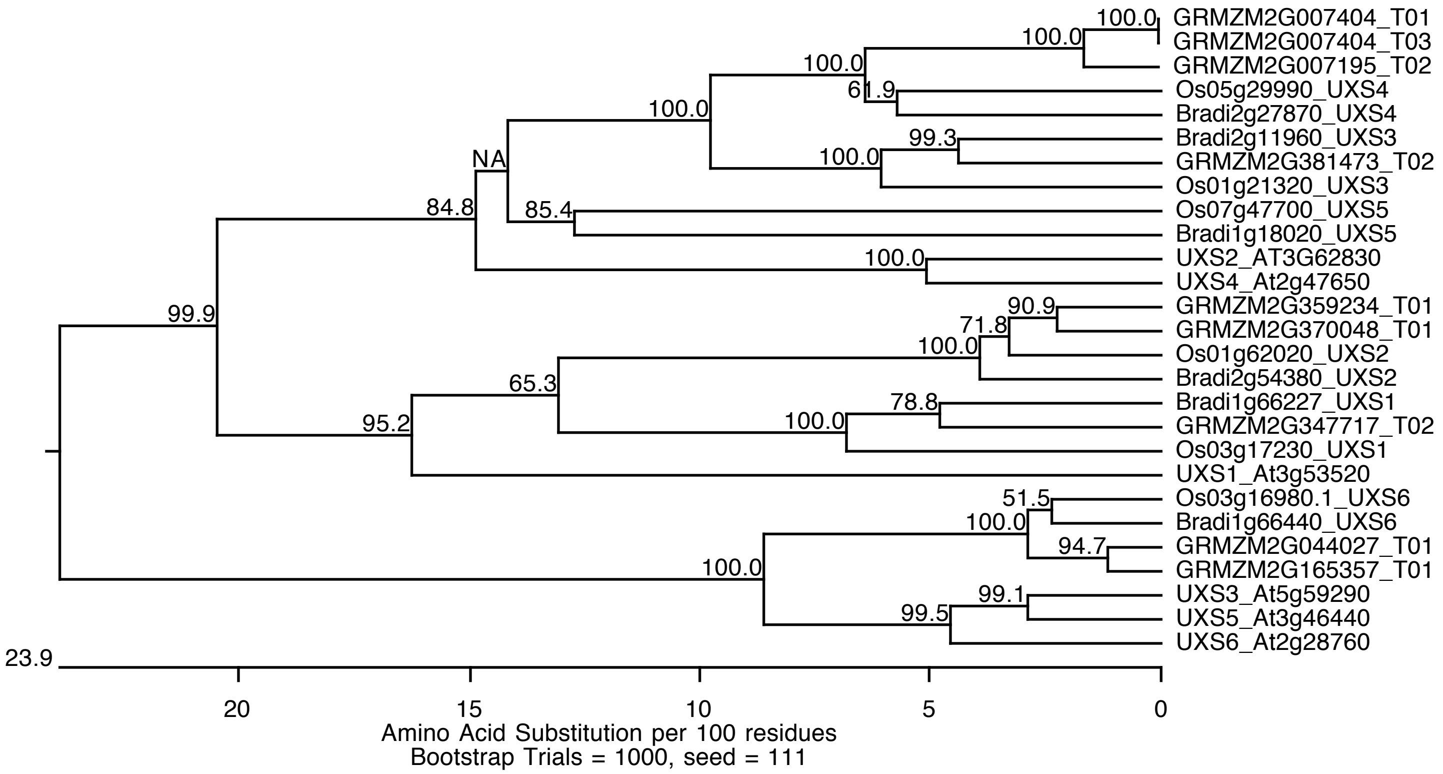

Supplemental Figure S3. UXS phylogenetic tree

Phylogenetic tree for predicted UXS enzyme sequences aligned in Sup. Fig. S2.

Supplemental Figure S4.

A

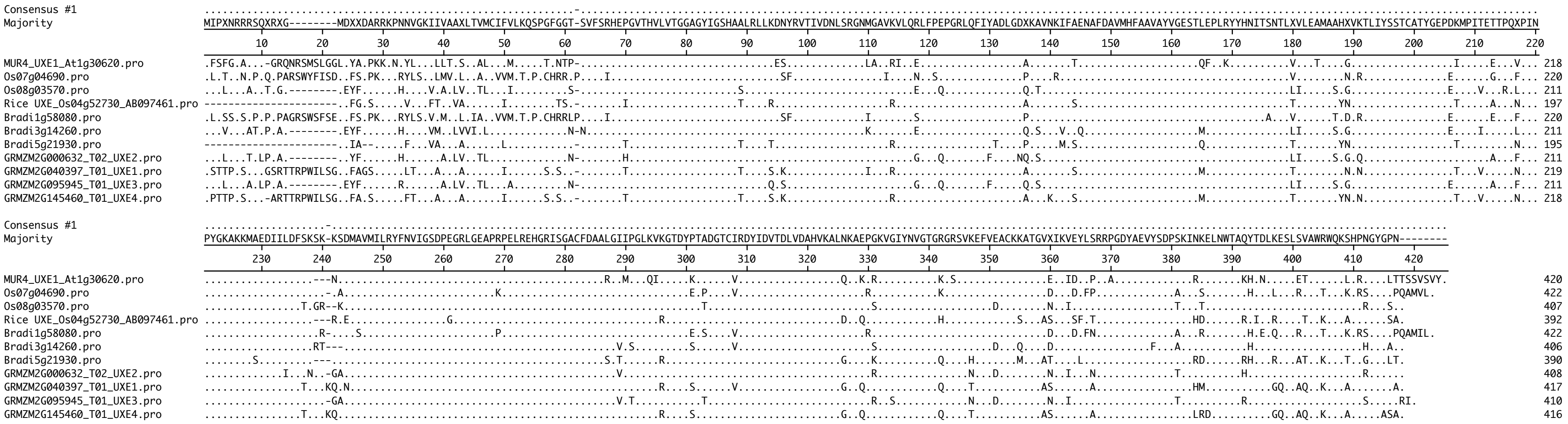

B

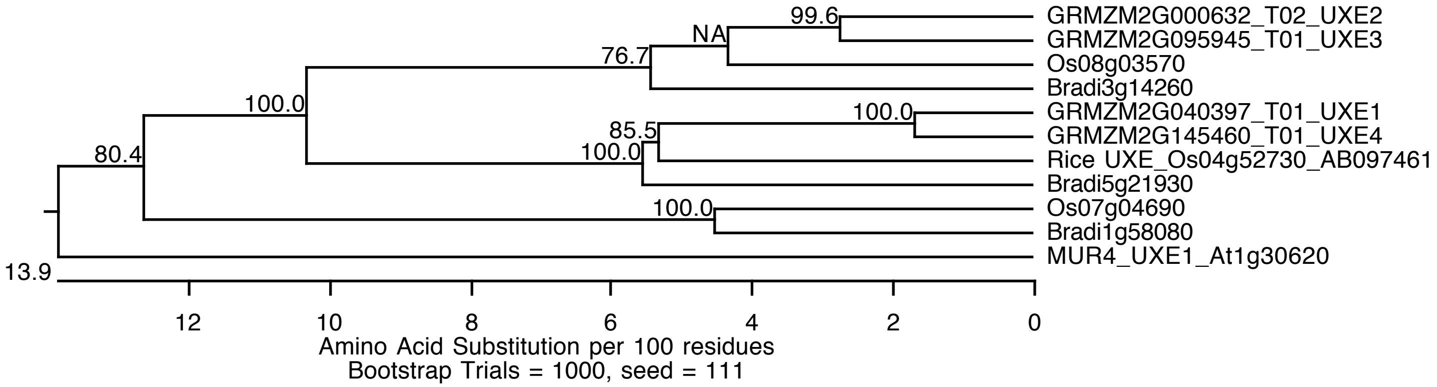

Supplemental Figure S4. UXE protein sequence alignment and phylogenetic tree

(A) Protein alignment of predicted UXE enzyme sequences for *Arabidopsis thaliana*, rice, *Brachypodium distachyon* Bd21, and maize. Majority consensus sequence is on top. Amino acid deviations from the majority consensus sequence are indicated for each protein sequence. (B) Phylogenetic tree for predicted UXE enzyme sequences aligned in (A).

Supplemental Figure S5.

A

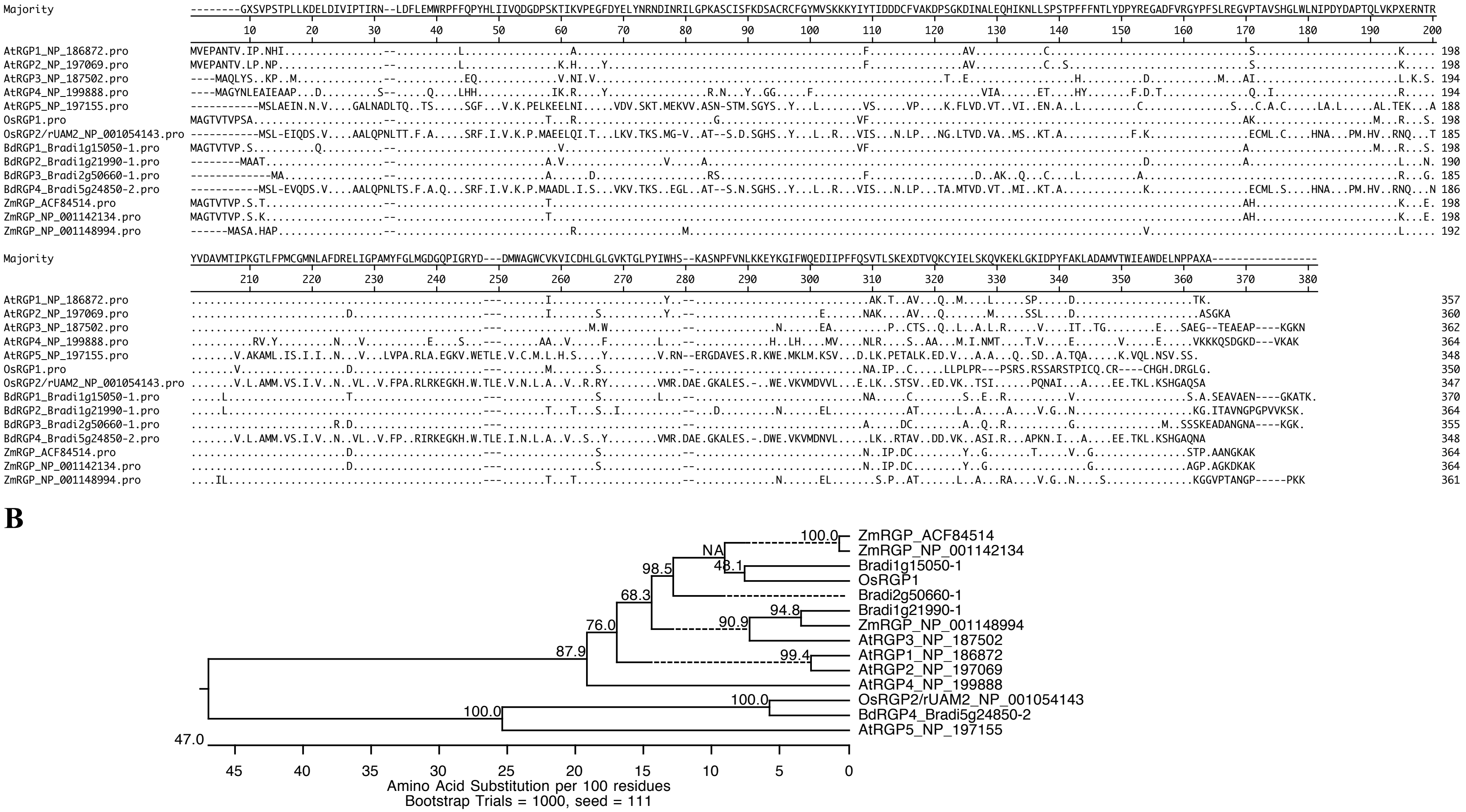

Supplemental Figure S5. RGP/UAM protein sequence alignment and phylogenetic tree
(A) Protein alignment of predicted RGP enzyme sequences for *Arabidopsis thaliana*, rice, *Brachypodium distachyon* Bd21, and maize. Majority consensus sequence is on top. Amino acid deviations from the majority consensus sequence are indicated for each protein sequence. (B) Phylogenetic tree for predicted RGP enzyme sequences aligned in (A).

Supplemental Figure S6.

A

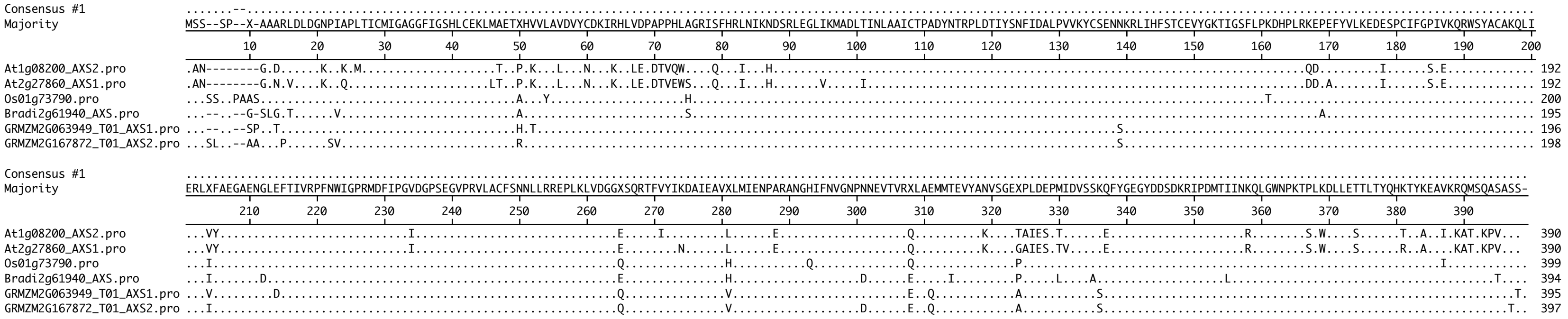

B

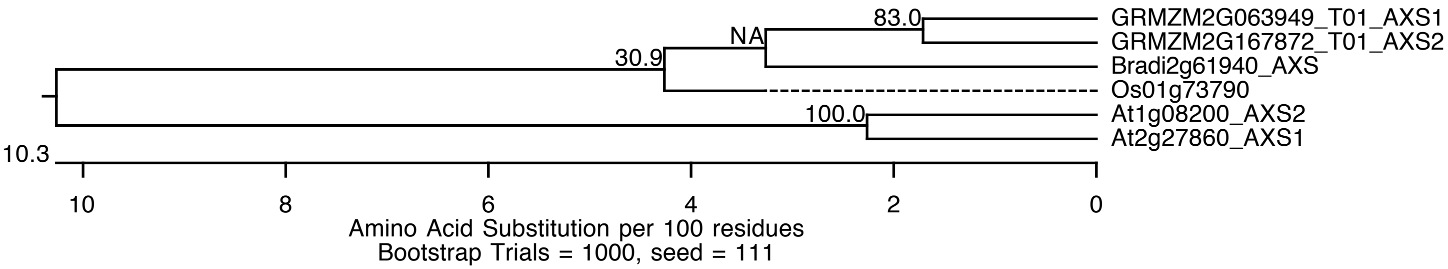

Supplemental Figure S6. AXS protein sequence alignment and phylogenetic tree
(A) Protein alignment of predicted AXS enzyme sequences for *Arabidopsis thaliana*, rice, *Brachypodium distachyon* Bd21, and maize. Majority consensus sequence is on top. Amino acid deviations from the majority consensus sequence are indicated for each protein sequence. (B) Phylogenetic tree for predicted AXS enzyme sequences aligned in (A).

Supplemental Figure S7. Cloned cDNA sequences

FASTA formatted sequences for cDNAs used in this study. Upper case indicates putative coding sequence. Lower case text indicates UTRs (5' or 3') contained within clones. Black sequences were cloned and sequence verified. Blue sequences were derived from predictions based on the *Brachypodium distachyon* Bd21 genome sequence and were used for primer design and bioinformatics.

```
>Bradi1g08120_UGD1
ataagcagacaagATGGTGAAGATCTGCTGCATCGGAGCTGGCTATGTCGGCGGCCCAACAATGGCTGTCATTGCCATCAAGTGCCCAGCAATTGAG
GTTGTTGTTGTTGACATCTCCAAGCCTCGCATTGATGCCTGGAACAGCGACGTGCTCCCAATCTACGAGCCTGGTCTCGATGATGTTGTGAAGGCGT
GCAGGGGCAAGAACCTCTTCTTCAGCACTGATGTTGAGAAGCACGTCGCCGAGGCCGACATCATCTTTGTGTCAGTGAACACCCCCACCAAGACCCG
TGGTCTTGGAGCTGGCAAGGCTGCCGACCTCACCTACTGGGAGAGCGCTGCCCGGATGATCGCTGATGTCTCCAAATCTGACAAGATCGTCGTTGAG
AAGTCCACCGTCCCTGTCAAGACTGCAGAGGCCATTGAGAAGATCTTGACCCACAACAGCAAGGGCATCAACTACCAGATCCTCTCCAACCCGGAGT
TCCTCGCTGAGGGAACAGCGATCGATGACCTGTTCAAGCCTGACAGAGTGCTCATCGGTGGCCGGGAGACACCTGAGGGCAGGAAGGCCGTTCAGGC
CCTCAAGGAGGTGTACGCCCACTGGGTTCCTGAGGAGAACATCATCACCACCAACCTGTGGTCTGCTGAGCTCTCCAAGCTCGCTGCCAACGCTTTC
CTGGCCCAGAGGATCTCCTCTGTGAATGCCATGTCCGCGCTCTGCGAGGCCACCGGCGCCAACGTTTCCGAGGTGTCTTACGCCATCGGGAAGGACT
CCAGGATCGGCCCCAAGTTCCTGAACGCCAGCGTTGGGTTCGGCGGGTCCTGTTTCCAGAAGGACATCCTGAACCTGGTGTACATCTGCGAGTGCAA
CGGCCTCCCCGAGGTGGCCAACTACTGGAAGCAGGTCATCAAGATCAACGACTACCAGAAGAGCCGCTTCGTCAACCGCGTCGTGTCCTCCATGTTC
AACACCGTCTCCGGCAAGAAGATCGCCGTCCTCGGCTTCGCCTTCAAGAAGGACACCGGTGACACCAGGGAGACCCCAGCCATTGACGTGTGCAAGG
GTCTTCTGGGTGACAAAGCCCAGGTCAGCATCTACGACCCCCAGGTGACTGAGGACCAGATCCAGAGGGACCTGGCCATGAACAAGTTCGACTGGGA
CCACCCGATGCACCTGCAGCCGACCAGCCCCACCGCCGTGAAGCAGGTGAGCGTGGTCTGGGACGCGTACGAGGCCACCAAGGGTGCCCACGGCGTC
TGCATCCTCACCGAGTGGAACGAGTTCAAGACCCTGGACTACCAGAAGATCTTCGACAACATGCAGAAGCCTGCCTTCATCTTCGACGGCCGCAACG
TCGTCGACGCCGAGAAGCTCAGGGAGATCGGCTTCATCGTCTACTCCATCGGCAAGCCGCTCGACGGGTGGCTCAAAGACATGCCCGCGGTGGCTTA
Attctctcgtcggagctgcaggccagggggtcgacaaggaacagagaagtggtttgaccattctttatagtttgcttttgcaggagacgctacatt
ttctctcgggcataaaattgggttccgtctcaagttttcaatgtttgctgcaccagacgtctgtagatcttataattaatcctcattccttgtgctg
tctgaggaacacccggacatttcttttgtatccagtgaactttgtagccggttgattttcaaccgttttgctgaaatcattacagaatcgttgtatt
tcctccgaaatctacattgttcgttgctcttattttcat
```

```
>Bradi4g25140_UGD2
tccgcgacgacccaggaggagcagcagcctcctgcttgcttcccgccgccgccgccgcggcagaggtgcccgcctacaggcATGATGGTGAAGATCT
GCTGCCTTGGAGCTGGCTATGTCGGTGGCCCGACAATGGCGGTCATCGCCCTCAAGTGCCCTGACATTCAGGTGGTTGTGGTCGATATCACCAAGTC
TCGTATTGACGCTTGGAACAGTGACACACTCCCAATCTACGAGCCTGGTCTTGATGATGTCGTGAAGCAGTGCCGTGGGAGGAACCTCTTCTTCAGC
AATGACATTGAGAAGCATGTTTGTGATGCTGACATCATCTTTGTGTCAGTAAACACCCCAACCAAGACCCGGGGCCTTGGAGCTGGCAAGGCCGCTG
ATCTTACCTACTGGGAAAGTGCAGCTCGCATGATTGCTGATGTGGCCAAATCTGACAAGATTGTGGTCGAGAAGTCCACTGTTCCAGTCAAGACTGC
TGAGGCTATTGAGAAGATCTTGACGCACAACAGCAATGGCATCAACTTCCAGATCCTCTCCAACCCAGAGTTCCTTGCTGAGGGTACTGCCATCCAG
GACCTATTTAACCCTGACCGTGTTCTTATCGGAGGACGGGAGACTCCGGAAGGCCAAAAGGCTGTTCAGACACTGAAAGCTGTGTATGCTCATTGGG
TTCCGGAGGATCAGATCCTGACTACCAACTTGTGGTCTGCGGAGCTGTCGAAGCTTGCAGCTAATGCATTCTTGGCCCAGAGGATCTCCTCAGTGAA
TGCCATGTCAGCCCTGTGCGAGGCCACTGGTGCGAATGTTTCTGAGGTTTCTTATGCTGTGGGCAAGGACTCCAGGATTGGGCCAAAGTTCCTGAAT
GCAAGTGTTGGATTTGGAGGCTCTTGCTTCCAGAAGGATATTCTCAACCTGGTTTACATCTGCGAGTGCAATGGCCTCCCAGAAGTGGCCAACTACT
GGAAGCAGGTCATCAAGATCAATGACTACCAGAAGAGCCGGTTCGTGAACCGTGTTGTTTCCTCTATGTTCAACACCGTCGCGAACAAAAAGATTGC
TGTGCTTGGTTTTGCATTCAAGAAGGACACCGGTGACACAAGGGAGACTCCTGCTATCGATGTCTGCAAGGGTCTGCTGGGTGACAAGGCTAAGATC
AGCATTTATGATCCTCAAGTGACTGAGGATCAAATCCAGCGTGACCTTGCAATGAACAAATTTGATTGGGACCACCCTGTTCATCTCCAGCCAATGA
GCCCCACAACCACCAAGCAAGTTTCAGTTACCTGGGATCCATATGAGGCGGCCAAGGATGCCCATGGCATCTGCATCCTGACTGAGTGGGATGAATT
CAAGAAACTGGACTACCAAAGGATCTATGAGAGCATGCAGAAGCCCGCCTTCGTTTTCGATGGCCGCAACGTTGTCGACTCTGAGAAGCTCAGGGAG
ATTGGCTTCATTGTCTACTCCATTGGCAAGCCACTTGATGCTTGGCTCAAGGACATGCCCGCTGTCGCTTAAatgaactctgatcagaaggaatcca
tgagcagcagctattgatagAggcccagcttgttattattattcgttatagttgctatttatcatgtgccagcaaattcagaatgtcattctttgct
tccttgttttaaaagtcttctctttctttttctttttggggtttacagtcttagaatggcaggcatggtcactgggatgtgtcttcattcctctgct
ccgttgtcacagctttgtagcagtgtctgttgttgcgttcctttcat
```

```
>Bradi1g10650_UGD3
gttaagcggacaagATGGTGAAGATCTGCTGCATCGGAGCTGGCTATGTCGGCGGCCCAACGATGGCTGTCATTGCCGTCAAGTGCCCAGCAATTGA
GGTTGTAGTTGTTGACATTTCCAAACCTCGCATTGACGCTTGGAACAGCGACCACCTCCCAATCTACGAGCCTGGTCTTGATGAAGTCGTGAAGGAG
TGCAGGGGCAGGAACCTCTTCTTCAGCACTGAAGTCGAGAAGCATGTTGCTGAGGCTGACATCATCTTTGTATCAGTGAACACCCCTACCAAGACCC
GTGGTCTTGGTGCAGGCAAGGCTGCCGACCTCACCTACTGGGAGAGTGCTGCTCGGATGATTGCTGATGTCTCCAAGTCTGACAAGATTGTCGTTGA
GAAGTCTACTGTCCCTGTCAAGACTGCAGAGGCTATTGAGAAGATCTTGACCCACAACAGCAAGGGTATCAACTACCAGATCCTCTCAAACCCAGAA
TTCCTTGCGGAGGGCACGGCGATCGAAGACCTATTCAAGCCTGACAGAGTGCTCATTGGTGGCCGGGAGACGCCTGAGGGCAGGAAGGCTGTTCAGG
CTCTCAAGGAGGTTTATGCTCACTGGGTTCCTGAGGACCAGATCATCACCACCAACCTGTGGTCCGCTGAGCTCTCAAAGCTCGCGGCCAATGCATT
CTTAGCACAGAGGATTTCCTCTGTTAATGCCATGTCTGCGCTCTGTGAAGCCACTGGTGCCAATGTGTCTGAGGTATCTTACGCCGTGGGTAAGGAC
```

TCCAGGATTGGCCCCAAGTTCCTAAATGCCAGTGTGGGGTTTGGGGGCTCCTGCTTCCAGAAGGATATCTTGAACCTCGTGTACATCTGCGAGTGCA
ATGGCCTCCCTGAGGTGGCCAGCTACTGGAAGCAGGTCATCAAGATCAATGATTACCAGAAGAGCCGGTTTGTCAACCGTGTTGTGTCCTCCATGTT
CAACACAGTCTCTGGCAAGAAGATCGCCATTCTCGGTTTCGCCTTCAAGAAGGACACCGGTGACACCAGGGAGACCCCAGCGATCGATGTGTGCAAG
GGCCTGTTGGGTGACAAGGCTCAGGTCAGCATCTATGATCCCCAGGTTACTGAGGAGCAAATCCAGTGGGATCTGGCCATGAACAAGTTTGACTGGG
ACCACCCGACGCACCTACAGCCGACAAGCCCCACGGCCATGAAGCAGGTGAGCGTTGTCCGGGGTGATGCATACGAGGCCACCAAGGGGGCCCATGG
AATCTGCATCCTCACTGAGTGGGAAGAGTTCAAGACTTTGGACTACCAGAAGATTTTTGACAACATGCAGAAGCCAGCCTTTGTCTTCGATGGGCGC
AACGTTGTGGATGCCGAGAAGCTCAGGGAGATTGGCTTCATCGTCTACTCCATTGGCAAGCCGCTTGATGCGTGGCTCAAGGACATGCCTGCTGTTG
CTTAAttgtgcacagttcacttgaaagtgcaccaccggagatgcaagagtttgcttttgcaggagacggtatattttctgtcaggcataaaatttgg
ttagtatgtttgtcttgagttcttcatgcttgtattgccatctgtagattatacgtacctcagtccatgggatgtttgtgtctgaggaatgtaacga
tgtttctttgtatccaatgagctttatagctggtccaactgtctcaagtttttctccccgtaa

>Bradi2g54380_UXS2
ATGAAGCAGCTCCACCACAAGTCCCCCACCCACGCGCCGTCGCCGGCGCACGCGCCGGCGTCCAAGACCTCCAAGCCCGCGCGCCCCGGACCGCGCA
CGTGGGTCGGCTACCTTCTCCTCGAACAGCGCCTCCTCTTCGTCCTCCTCGGCGCGCTCATAGCCACCTCCTTCTTCCTCCTCCGGCCCTACATCTT
CTCCCTATCCCCCTCCAACGTCACCGACCGCAGCCCCATATTTTCCTTCGCGGCTCGCTCGTCCGCCTCCCGCGTCCCCGCGGGCTTCCGCCCGCCG
CCTCGCCGCGTCGTCGTCACCGGCGGGGCGGGCTTCGTGGGCAGCCACCTCGTGGACAGGCTGCTGGAGCAAGGGGACAGCGTGATCGTGGTGGACA
ACTTCTTCACCGGGAGGAAGGAGAACGTTGCGCACCACCTACGGAACCCCAGATTCGAGCTGCTCCGCCACGACGTCGTCGAGCCCATCCTTCTCGA
GGTCGACCGGATCTACCACCTCGCGTGCCCCGCATCGCCCGTGCACTACAAGTACAACCCCATCAAGACCATCAAGACAAATGTCATGGGAACTTTG
AATATGTTGGGTTTGGCAAAGCGAATTGGTGCAAGGTTCTTGTTGACTAGCACAAGTGAAGTTTATGGTGATCCACTTGAGCATCCACAAAAGGAAA
CTTATTGGGGGCATGTTAATCCTATAGGTGTTAGGAGTTGTTATGATGAGGGCAAAAGAACAGCAGAGACTTTGACCATGGACTATCATCGTGGTGG
TGGTGTTGCGGTACGCATTGCCCGCATCTTCAACACATATGGTCCTCGTATGTGCCTAGATGATGGCCGTGTGGTTAGCAATTTTGTTGCACAGGCA
CTGCGTAGACAACCAATGACAGTTTATGGTGACGGAAAACAAACTCGAAGTTTTCAATATGTTTCTGATCTGGTTGCAGGATTGATGGCTCTCATGG
AGAGTGATCACATTGGTCCTTTCAATCTGGGAAATCCAGGAGAGTTCACCATGTTGGAACTTGCGGAGGTTGTGAAGCAAACAATTGATCCAATGTC
GACAATTGAATTCAAACCCAATACAGCTGATGATCCCCATATGAGAAAGCCAGATATTACCAAGGCCAAGCAACTGCTAGGTTGGGAGCCAAAGGTG
TCGCTCAAGGAAGGCCTTCCCTTAATGGTGACAGATTTCCGTCAAAGGATCTTGGATGAGTAAacaaagaaacatagtgacacatcggttatccatt
tgattctgaatgctgctgcaaatacatgccgaagttcttcgccaactggatccatgcagtagaatcgtttatttacatcttggtgagcctgaacaga
accagtcacatattttgtccacgtgtggagctttagaagtggactggaagataggggaaggagttgtatttcgggagacattgggtttcagtctttc
ggtta

>Bradi2g11960_UXS3
ATGGCGTCGGAGCTAACGTACCGCGGCGGGGCAGCCACGGGCTCCGCCTCCGACGGCGGGGAGTACTCTCCCAAGCCCTCCAAGCCGCTCTCCTGGC
TCACCCGCGCCGCCCGCTACGCCACCGCCGAGCACCGGCCCCTCTTCGCCCTCGTCGGCATGCTCTTCGCCGCCGCCCTCTTCACCTTCTCCTCGTC
TTCCTCCTCGGCGGGATACACCTCCCCCTCCGGCCCCGCCGCCGTCTCCTTCAAGCATCTCGCCAACATCGCGCACCCGTCGCTGCACGAGTCCGTC
GGCGGCAAGATGCCCCTGGGGCTGCGCCGGCGCGGGCTACGGGTCCTCGTGACTGGCGGCGCCGGGTTCGTGGGGAGCCACCTGGTGGATCGGCTCA
TGGAGCGCGGCGACAGCGTGATCGTGGTGGACAATTTCTTCACGGGGCGCAAGGGGAACGTCGCGCACCACCTCGGGAACCCCAGGTTCGAGGTCAT
CCGGCACGATGTCGTCGAGCCCATACTGCTCGAGGTCGACCAGATCTACCACCTCGCTTGCCCTGCCAGCCCCGTGCACTACAAGTACAACCCAATC
AAGACAATCAAGACCAATGTGGTCGGGACACTGAACATGCTTGGATTGGCAAAGAGGATTGGTGCAAGGTTTCTCCTCACCAGCACAAGTGAAGTCT
ATGGTGATCCCCTCCAGCACCCTCAGGTGGAGACTTACTGGGGCAATGTCAATCCCATTGGTGTCAGGAGTTGCTATGATGAGGGTAAACGCACAGC
TGAAACACTGACCATGGATTACCACCGTGGTGCCAACCTTGAGGTCAGGATTGCTCGAATTTTTAACACGTATGGTCCTCGCATGTGCATTGATGAT
GGCCGTGTTGTCAGTAACTTTGTTGCTCAGGCACTGAGGAAGGAGCCTTTGACTGTCTACGGTGATGGCAAGCAGACTAGGAGCTTTCAATACGTCT
CTGATCTGGTGGAAGGGTTGATGAAACTGATGGAAGGGGAGCACATAGGACCATTCAACCTGGGTAACCCTGGTGAGTTCACCATGCTGGAGCTGGC
TAAGGTGGTACAGGACACCATTGACTCGAGTGCAAGCATTGAGTACCGGCCAAATACCGCTGATGATCCACATAAGCGCAAGCCAGACATTACCCGC
GCAAAAGAACTCCTGGGTTGGGAGCCCAAGGTCCCTCTACATGAGGGGCTTCCCCTCATGGTCACCGACTTCCGCAAACGTATCTTTGGGGATCAAG
AGGAGTCGACTACCACAGCTGGTGGCATGTCTTAA

> Bradi2g27870_UXS4
ATGGCGTCGGAGCTCACCTACCGAGGCGGGGCGGCGTCCCCCTCCGCCGCCGGTGCCGGTGCCGCCGGGTACGCCCCGAAGCCCTCTAATTCGAAGC
CGCTCGCGTGGCTCCCCCGCGCCGCCCGGTACGCCGCCGCCGAGCACCGCCCGCTCTTCGCCCTCGTCGGGATGCTCTTCGCCGCCGCCGTCTTCTG
CATCGCTGCCCCCTCCTCCTCCTCCGCCCCCGCCACCGCATCCTCCTACTCCTCCGGCGCCCGCGCCGTCACCAACCCGCTCGCCCGCTTCTCCCTG
GACTCGTCGtCGGCGGCCGCGCACCACCCCGCCCGCCACTTCGTCGGCGGCAAGGTGCCCCTGGGCCTCAAGCGGAAGGGCCTCCGCGTCCTCGTCA
CCGGCGGCGCCGGCTTCGTGGGGAGCCACCTCGTGGATCGCCTCGTGGAGCGCGGCGACAGCGTCATCGTCGTCGACAACCTCTTCACGGGCCGCAA
GGAGAACGTGGTCCACCACTTCGGCAACCCCAACTTCGAGATGATCCGCCACGACGTCGTCGAGCCCATCCTCCTCGAGGTCGACCAGATCTACCAT
CTCGCCTGCCCCGCCTCCCCCGTCCACTACAAGTACAACCCCGTCAAGACAATCAAGACCAATGTGGTTGGTACATTGAACATGCTTGGATTGGCGA
AGAGGATCGGAGCCAGGTTCCTCCTCACCAGCACCAGTGAGGTCTACGGTGATCCCCTCCAGCACCCTCAGGTGGAGACTTACTGGGGCAATGTCAA
CCCTATCGGTGTCAGGAGCTGCTACGATGAGGGCAAGCGTACAGCTGAAACATTGACCATGGACTACCACCGTGGTGCCAACCTTGAGGTCAGGATT
GCTCGGATCTTCAACACATATGGTCCTCGCATGTGCATTGATGACGGTCGTGTTGTTAGCAACTTTGTTGCTCAGGCGCTAAGGAAGGAGCCTTTGA
CGGTTTATGGTGACGGCAAGCAGACCAGGAGCTTCCAATACGTTTCTGATCTGGTTGAGGGGTTGATGAGGCTGATGGAAGGGGAGCACGTAGGGCC
ATTCAACTTGGGTAACCCTGGTGAGTTCACCATGCTGGAGCTGGCCAAGGTTGTCCAGGACACCATTGATCCGAATGCGCGGATTGAGCACCGTGAG
AACACTCAGGACGATCCTCACAAGCGCAAGCCAGACATCAACCGTGCCAAGGAGCTCCTCGGCTGGGAGCCGAAGATCCCCCTCCGTGAGGGCCTTC
CTCTCATGGTCACTGATTTCCGCAAGCGCATCTTTGGCGACCAAGATAGCGCCACCACAGCCACCGGAGGCCAATAAggggtgtagaagggacttga

agactttgaaattttggtggcctgcttctagtttctcggttgatctccctactgacagagctatggccacgtagttatcgaaagctttactaaaata
ccttggttgttggtgtaatgtcatttttttttctgaattgtcttcccctcctcggtctcagct

>Bradi1g18020_UXS5
ATGGCGTCCTCCGAGCTCACCTACCGCGGCCAGCAGCACGACGCCCAGCAGCCAGCACCCCTTGACGACAACAACAACAACAAGCCACGGAAGCCGA
CGATGCCGATGCCGGCCCTGCGGTACGTGCTGGGCGAGCAGCGCCTTGCCTTCGCCATGGCCGGCATGGCCCTCGCCACCCTCTTCTTCCTCCTCCT
CTCCCCTTCCTCCACCCCCCACTCCTCCACCACGGCCGCCGTCTCAAACGTGGCCCACCTGGCAGCGGTGGGCCTCGCCTCCCACCACTCCCTCTCC
GGCGCCACCACCACGCGCATGCCGATGCCGACATCGGCATGGCCACGGCTCCCAGGGCGCGTCCCGCTGGCCCTGAAGCGCAAAGGCCTGCGCGTCC
TCGTCACGGGCGGCGCCGGCTTCGTGGGCAGCCACCTGGTGGACCGCCTGCTGTCCCGGCCGGAAGTCACCAGCGTCATCGTGGTGGACAACTTCTT
CACGGGGCAGAAGTCCAACGTGGCGCACCACGTGGCCTCCGGCGACCCGAGGTTCGAGCTGATCAGGCACGACGTGGTGGAGCCGATCCTGCTGGAG
GTGGACCAGATCTACCACCTGGCGTGCCCGGCGTCCCCCGTGCACTACAAGTTTAACCCCGTGAAAACCATCAAGACCAACGTCGTCGGGACGCTCA
ATATGTTGGGGCTGGCGAAACGGGTTGGGGCGAGGTTCTTGTTGACGAGTACCAGTGAGGTTTATGGGGATCCTTTGCAGCATCCTCAGGTTGAGAC
TTACTGGGGCAACGTCAATCCCATCGGTGTGAGGAGTTGCTACGACGAGGGGAAGCGGACGGCGGAGACGCTGACCATGGACTACCACCGCGGCGCC
AACCTCGAGGTGAGGATTGCCCGGATCTTCAACACCTACGGGCCACGCATGTGCATCGACGATGGCCGGGTCGTCAGCAACTTCGTCGCTCAGGCGC
TGAGAAAGGAACCACTGACGGTGTACGGGGACGGCAAGCAGACCAGGAGCTTCCAGTACGTCTCCGACCTGGTGGAGGGTCTGATAAAGCTGATGGA
AGGGGAGCACGTGGGCCCGTTCAACCTGGGGAACCCGGGGGAGTTCACGATGCTGGAGCTGGCCAAGGTGGTGCAGGACGTCATCGACCCAAACGCC
CGGATCGAGTTCCGGCCCAACACCGCCGACGACCCGCACAAGCGCAAGCCCGACATCACGCGGGCCAAGGACCTCCTGGGCTGGGAGCCCAAGGTGC
CCCTCGCCAAGGGCCTCCCGCTCATGGTCAACGACTTCCGCTCCCGCATCTTCGGCGACCAGATCAAGCCTGACGCCGCCAATTAA

>Bradi1g66440_UXS6
gtccaaactaagcccgaatcctcccagcggccagcgccaccgccgcccctggtgttggacgcgATGGCGCAGAAGGAGGCCAATGGCAACGGCGCC
ACCACACGCCCGCCGCCGACGCCCTCACCGCTCCGCTTCTCCAAGTTCTTCCAGGCCAACATGCGGATCCTGGTCACCGGCGGAGCTGGGTTCATCG
GCTCGCACCTCGTCGACAAGCTCATGGAGAACGAGAAGAACGAGGTCATTGTTGCTGATAACTTCTTCACTGGTTCAAAAGACAACCTGAAGAAGTG
GATCGGTCACCCAAGATTTGAGCTCATCCGTCATGATGTCACGGAACCACTGCTGTTGGAGGTTGATCAGATCTATCACCTTGCTTGCCCGGCTTCA
CCAATCTTCTACAAGCACAACCCTGTGAAGACTATCAAGACAAATGTTATTGGAACCTTGAACATGCTTGGACTTGCAAAGAGAGTTGGTGCTAGAA
TTTTGTTGACTTCGACCTCTGAAGTTTATGGTGATCCTCTGGAGCACCCTCAGACCGAGGCCTACTGGGGCAATGTTAACCCAATTGGAGTTAGGAG
CTGCTACGATGAGGGTAAGCGGGTAGCCGAGACACTGATGTTTGACTACCACAGGCAGCATGGCATTGAGATCCGTATTGCCAGGATTTTCAACACC
TATGGACCTAGGATGAACATTGATGATGGGCGTGTTGTTAGTAACTTCATTGCTCAGGCCATACGTGGTGAAGCCCTGACTGTCCAGAAGCCAGGAA
CACAGACTAGGAGCTTCTGCTATGTTGCTGATATGGTTAATGGTCTTATGAAGTTGATGAATGGAGACAACACTGGACCGATTAACATTGGAAACCC
TGGTGAATTTACCATGTTGGAACTTGCCGAGAATGTGAAGGAGTTGATCAACCCAGAAGTAACAGTAACTATGACTGAGAACACTCCTGATGACCCT
CGCCAGAGGAAGCCAGACATCACAAAGGCCAAGGAGGTTCTTGACTGGGAGCCCAAGGTCGTCCTGCGTGACGGCTTGGTGCTCATGGAAGATGATT
TCCGGGAGCGCCTGGCAGTGCCCAAGAAGAGCAAGGCCTAAgtttcgccccttggtatttggcgaacaatatcaccaggagcatactcatacatggg
tgggttcaccatgattcgttgttgccgtgttacggaatttgagttccaataaaccaattacaccttcttcgatcacttgaaagattgtattattaga
aactttattcatacggggctggctgtttgt

>Bradi5g21930_UXE1
tctccatcgccgtccccgttcttcctcggttgcggtaccaggtATGGACATCGCGAGACGCAAACCTAATTTCGTGGGAAAGGTCGCTGTGGCTGCC
GCCCTCACTGTCATGTGCATACTTGTATTGAAACAGTCTCCTGGTTTCGGCGGTACTAGTGTGTTCTCTCGCCATGAAACTGGGGTGACTCATGTGC
TGGTGACAGGAGGTGCTGGATACATCGGCTCGCATGCTACCCTTCGTCTCCTTACGGACAATTACCGAGTTACCATTGTGGATAACCTTTCTAGAGG
GAACATGGGGGCTGTCAGAGTTCTTCAACGGTTATTTCCAGAACCTGGGAGGCTTCAATTTATATACACTGATTTAGGCGATCCGAAAGCTGTGAAC
AAAATGTTTTCTGAGAACGCATTCGATGCTGTTATGCACTTTGCTGCTGTTGCTTATGTTGGTGAGAGCACGCAGGAGCCACTCAGGTACTACCACA
ACATAACATCAAATACTTTGACAGTGCTTGAGGCAATGGCAGCATATAATGTAAAAACTTTGATTTACTCGAGTACTTGCGCAACATATGGTGAGCC
TGACACAATGCCTATTACCGAAACAACTCCTCAGAATCCTATCAATCCATATGGGAAGGCAAAGAAGATGTCAGAGGACATAATTTTAGATTTCTCA
AAGAAATCGGACATGGCTGTCATGATCTTAAGATACTTCAATGTTATTGGATCAGACCCTGAGGGACGCCTTGGGGAAGCTCCGAGGCCAGAGTTGC
GTGAGCACGGAAGGATTTCTGGTGCTTGTTTCGATGCAGCATCAGGAACCATTCCAGGGCTAAAGGTTCGAGGAACTGACTACCCGACTGCTGATGG
AACTTGCATAAGGGACTACATAGATGTCACAGATCTTGTTGATGCTCATGTCAAAGCTCTTGGTAAAGCAGAGCCTAAAAAAGTTGGAATCTACAAT
GTTGGCACAGGGCAAGGTAGGTCGGTGCATGAGTTCGTAGAAGCATGCAAGATGGCGACCGGAGCTACCATCAAGGTCGAATTACTTTCCCGGAGAC
CAGGAGACTATGCTGAGGTTTACAGTGACCCATCCAAAATCCGCGACGAGCTGAACTGGACAGCTCGCCACACAGATCTTCGCGAAAGCCTTGCAAC
CGCATGGAAATGGCAGAAGACACACCCTGGCGGATATGGGTTGACCTGAttcagtgctcagtaggttcagattcgacggtgttcttccaacacaggt
tcatggatccttgtgcagtgatgcagccttcaggaaactggcccttcattcaatagacgctttgcaggtagccacagaagagtaatgtataacttgc
agcacctggaagaagatacagagaatgcctccccatcccttgcttagaatcatactataggttttcttttccgttgttttacatttcattgattttg
gcatccactggctgaagcatgatataataacacatacaaagttgtggtgtagagaacgagatcaatcttatattatgaactatgatatgaacctttta
cagtggtggatggcatgat

>Bradi1g58080_UXE2
ccgcgacctttccttccagttccactctcccttcctcttcctcagcccgagtgccgcggcgtacgcgtgtccaccttccttctgcaaaaggatcgga
gattcggagggctggcctcgcactagcactagctgtctgcgcctgtgtctgggagaggaaaagggagagggttgttggatctgactctctctcactc
gctgcgcctctctctcttttgcgcggccggcggcggattccagcctttcttcaaattcgcagcagggacagatcgATGCTGCCCAGCAGCAGGAG
CAGGCCGCAGCCCAGACCCGCCGGCAGATCTTGGTCCTTCTCAGAGATGGACTTCTCCGACCCCAAGCGCAAGCCGAGGTACCTCAGCAAGGTAATC
ATGGCCGCGCTGCTCATCGCCATGTGCGTCGTCATGCTCACCCAGCCGCCCTGCCATAGGAGGCTCCCCAGTGTGTTCTCCATCCACGAGCCTGGAG

TAACACATGTGCTGGTGACGGGTGGTGCTGGCTACATTGGTTCGCACGCTGCTCTTCGGTTGCTGAAAGACTCCTTCAGAGTCACCATTGTGGATAA
TCTTTCgAGAGGAAATATCGGGGCAGTCAAGGTTCTTCAGAGCTTGTTTCCTGAGCCTGGGCGACTGCAATTTATATATGCTGACTTAGGGGATCCA
AAAGCTGTTAATAAAATATTTGCAGAAAATGCATTTGATGCTGTCATGCACTTTGCCGCCGTCGCTTATGTGGGCGAGAGCACGCTTGAGCCTCTCA
GGTACTACCATAACATCACTGCAAATACTCTAGTTGTGCTAGAAGCCATGGCGACACACGATGTGAGAACTCTGATCTACTCTAGCACTTGTGCCAC
CTATGGTGAACCTGAGAAGATGCCTATCACTGAAGAAACTCCCCAGTTTCCAATCAACCCGTATGGTAAGGCCAAGAAAATGGCAGAGGATATCATT
TTGGACTTCTCCAAGTCCAGGAAATCAGACATGTCAGTGATGATTCTAAGATACTTCAATGTCATTGGTTCTGACCCAGAAGGCAGGCTGGGTGAGG
CTCCACCACCTGAATTGCGTGAGCATGGCCGTATATCTGGTGCATGCTTCGATGCAGCACTAGGaATAATCCCAGGTTTGAAGGTGAAAGGTACCGA
CTATGAGACGTCCGATGGCACTTGTGTAAGAGATTACATTGATGTCACTGATCTGGTTGACGCCCACGTGAAGGCGCTGAACAAGGCAGAAAGAGGC
AAAGTTGGCATATACAACGTTGGCACTGGAAGAGGTAGGTCAGTGAAGGAGTTTGTGGAAGCTTGCAAGAAGGCAACAGGGGTTGACATCAAGGTCG
ACTACTTCAATCGCCGCCCCGGTGACTACGCAGAAGTGTACAGCGACCCTGCAAAGATCAACCGTGAGCTGAATTGGACAGCACAGCACACGGAACT
CCAAGAGAGCCTCAGGGTCGCATGGACTTGGCAGAAGAAGCACCGGAGTGGCTACGGACCGCCTCAGGCTATGATTTTGTGAagaggtttactttac
catgtagcatgatctgttggctgaagatttaggtggatatattcctaggaatgttcctcaagggctgtactggctcaataa

>Bradi3g14260_UXE3
ATGATtCCTGTAAACAaGAGGGCAACTCAGCCTAGAGCTGGGATGGAGTACTTCGATGCTAGGCGCAAGCCACATAATGTCGGGAAAGTCATGGTGG
CCCTGGTTGTCATAGTGCTCTGTATATTTGTTCTGAAGCAATCTCCTGGTTTTGGTGGCAATAATGTGTTTTCTCGCCATGAACCTGGGGTTACCCA
TGTCTTGGTGACAGGAGGAGCTGGCTATATTGGTTCACATGCCtCATTACGTCTGTTAAAGGATAATTATCGAGTTACCATTGTGGATAATCTTTCT
AGAGGAAATAAGGGAGCAGTAAAGGTTCTCCAAGAATTGTTTCCGGAGCCTGGGAGACTTCAATTCATCTATGCTGATCTTGGGGATCAAAAATCTG
TCAACAAGaTATTTGCTCAAAATGCATTTGATGCTGTGATGCACTTTGCAGCTGTTGCgTACGTGGGTGAGAGTACAATGGAACCTCTTAGGTATTA
TCACAATATTACATCGAACACCTTACTGATTTTGGAGGCTATGGCTTCTCATGGAGTCAAGACCCTTATTTACTCTAGTACCTGTGCCACCTATGGA
GAACCCGAGAAGATGCCTATAATAGAAACGACACCTCAGTTGCCAATTAACCCCTATGGAAAAGCCAAGAAAATGGCAGAGGACATCATACTAGATT
TCTCGAAGAGAACGGATATGGCTGTCATGATTTTAAGATATTTCAATGTTATTGGATCAGACCCAGAGGGAAGATTAGGTGAAGCTCCTAGGCCTGA
ACTACGAGAGCATGGAAGGATCTCTGGGGCATGCTTTGATGCAGCATTAGGAGTCATTTCAGGATTGAAGGTTAAAGGAACAGATTATTCTACAGCT
GATGGAACCTGCGTAAGAGACTACATTGATGTCACgGATCTAGTAGATGCTCATGTGAAAGCACTCAACAAGGCAGAGCCTAGTAAAGTTGGCATTT
ACAATGTTGGCACTGGAAGAGGTCGTTCAGTTAAGGAGTTTGTGGATGCCTGCAAACAGGCAACTGGGGTTGACATCAAAGTAGAGTACCTCAGCAG
GCGGCCAGGAGACTATGCCGAAGTATTCAGTGACCCTGCAAAGATCAACAAGGAGCTAAACTGGACTGCGCAACATACCGACCTCAAGGAGAGCCTG
TCGGTCGCATGGAGATGGCAGAAGTCGCATCCGCATGGCTATGGGGCAAAC

>Bradi1g15050_RGP1
ATGGCGGGGACGGTGACTGTCCCCGGGTCGTCGGTGCCCTCCACGCCGCTGCTCAAGGACCAGCTGGACATCGTGATCCCGACGATCCGCAACCTCG
ACTTCCTCGAGATGTGGCGCCCCTTCTTCCAGCCGTACCACCTCATCATCGTGCAGGACGGCGACCCCAGCAAGGTCATCAAGGTGCCAGAGGGCTT
CGACTACGAGCTCTACAACCGGAACGACATCAACCGCATCCTCGGCCCCAAGGCCTCATGCATCTCCTTCAAGGACTCGGCCTGCCGCTGCTTCGGC
TACATGGTCTCCAAGAAGAAGTACGTCTTCACCATCGACGACGACTGCTTTGTTGCCAAGGACCCATCTGGCAAGGACATCAATGCTCTTGAGCAGC
ACATCAAGAACCTCCTTAGCCCATCCACCCCATTTTTCTTCAACACCCTGTACGACCCATACCGTGAAGGTGCTGACTTTGTTCGTGGGTACCCCTT
CAGCCTTAGGGAGGGTGCCCCCACTGCTGTTTCCCATGGCCTGTGGCTCAACATTCCTGACTATGATGCACCCACACAGATGGTCAAGCCTCGTGAG
AGGAACAGCAGGTATGTTGATGCTGTCCTGACTATTCCAAAGGGAACCCTTTTCCCCATGTGTGGCATGAACCTTGCCTTTGACCGTACTCTTATTG
GCCCTGCAATGTACTTTGGCCTCATGGGAGATGGCCAGCCTATTGGTCGCTACGACGACATGTGGGCTGGATGGTGTGTGAAGGTCATCTGCGACCA
CTTGAGCCTGGGAGTGAAGACTGGCCTCCCATACCTGTGGCACAGCAAGGCTAGCAACCCCTTTGTGAACTTGAAGAAGGAGTACAAGGGCATCTTC
TGGCAGGAGGACATCATCCCGTTCTTCCAGAACGCCACCCTGTCAAAGGAGTGCGACACTGTCCAGAAGTGCTACATCTCACTTTCAGAGCAGGTCA
GGGAGAAGCTTGGGAAGATTGACCCTTACTTTGTGAAGCTTGCTGATGCCATGGTCACCTGGATTGAGGCCTGGGATGAGCTGAACCCATCTGCTGC
TGCTTCTGAAGCTGTTGCTGAGAATGGGAAGGCGACCAAG

>Bradi1g21990_RGP2
ATGGCCGCCACGCCGTCGACGCCGCTGCTCAAGGACGAGCTGGACATCGTGATCCCGACGATCCGCAACCTCGACTTCCTCGAGATGTGGCGGCCCT
TCTTCCAGCCCTACCACCTCATCATCGTGCAGGACGGCGACCCCGCCAAGGTCATCAAGGTGCCCGAGGGCTTCGACTACGAGCTCTACAACCGCAA
CGACGTCAACCGCATCCTCGGCGCCAAGGCCTCCTGCATCTCCTTCAAGGACTCCGCCTGCCGCTGCTTCGGCTACATGGTCTCCAAGAAGAAGTAC
ATCTACACCATCGACGACGACTGCTTCGTTGCTAAGGATCCATCAGGAAAGGACATAAATGCACTTGAGCAGCACATCAAGAACCTTCTGAGCCCTT
CTACTCCGTTTTTCTTCAACACTCTGTACGACCCTTACCGTGATGGTGCTGATTTTGTTCGTGGGTACCCCTTCAGCCTTCGTGAGGGTGCCCCGAC
TGCTGTTTCTCATGGGCTCTGGCTCAATATTCCTGACTATGATGCCCCTACCCAGCTTGTCAAGCCGCTAGAGAGAAATAACAGGTATGTTGATGCT
GTTCTTACAATCCCAAAGGGCACCTTGTTTCCGATGTGCGGGATGAACCTTGCATTTGACCGTGAGCTCATTGGTCCTGCAATGTACTTTGGCCTTA
TGGGTGATGGCCAGCCTATTGGTCGCTACGATGATATGTGGGCAGGATGGTGCACCAAGGTGATTACTGATCATTTGAGCCTGGGTATTAAGACTGG
TCTGCCCTACATCTGGCACAGCAAAGCAAGCGACCCATTTGTGAACCTGAAGAAGGAATACAATGGCATCTTCTGGCAAGAGGAGCTGATCCCCTTC
TTCCAGTCTGTGACTCTTTCCAAGGAGGCCACCACTGTTCAGAAGTGCTACCTCGAGCTGGCCAAGCAGGTGAAGGCGAAGCTTGGCAAGGTGGACG
GCTACTTTAACAAGCTCGCTGATGCCATGGTCACATGGATCGAGGCGTGGGACGAGCTTAACCCACCAAAGGGCGCAATTACCGCTGTGAACGGCCC
TGGCCCTGTCGTCAAGAGCAAGTGA

>Bradi2g50660_RGP3
ATGGCGCCGCTGCTCAAGGACGAGCTGGACATCGTGATCCCGACGATCCGGAACCTGGACTTCCTCGAGATGTGGCGTCCCTTCTTCCAGCCGTACC
ACCTCATCATCGTGCAGGACGGCGACCCGGCCAAGACGATCAAGGTCCCCGACGGCTTCGACTACGAGCTCTACAACCGCAACGACATCAACCGGAT
CCTCGGCCCCAGGTCCTCCTGCATCTCCTTCAAGGACTCCGCCTGCCGCTGCTTCGGCTACATGGTCTCCAAGAAGAAGTACATCTTCACCATCGAC

GACGACTGCTTCGTGGCCAAGGACCCGTCGGGGAAGGACATCGACGCGCTGGCGAAGCACATCCAGAACCTGCTCTGCCCATCCACGCCGCTCTTCT
TCAACACGTTGTACGACCCTTACGCGGAGGGCGCCGACTTCGTGCGCGGGTACCCGTTCAGCCTCCGGGAAGGGGTTCCGACGGCCGTGTCCCATGG
GCTCTGGCTCAACATCCCCGACTACGACGCCCCCACGCAGCTGGTCAAGCCCCGGGAGCGGAACGGCAGGTACGTCGACGCCGTCATGACCATCCCC
AAGGGCACCCTCTTCCCCATGTGCGGCATGAACCTCGCCTTCCGCCGCGACCTCATCGGCCCCGCCATGTACTTCGGCCTCATGGGCGACGGCCAGC
CCATCGGCCGCTACGACGACATGTGGGCCGGCTGGTGCGTCAAGGTGATCTGCGACCACCTGGGGTTGGGAGTCAAGACGGGGCTGCCCTACATCTG
GCACAGCAAGGCCAGCAACCCGTTCGTGAACCTCAAGAAGGAGTACAAGGGCATCTTCTGGCAGGAAGACATCATCCCGTTCTTCCAGGCCGTGACG
CTGTCCAAGGACTGCGACACCGTGCAGAAGTGCTACATCGCGCTCTCGCAACAGGTCAGGGAGAAGCTCGGCAAGATCGACCCTTACTTCGGCAAGC
TCGCCGACGCCATGGTCACATGGATCGAGGCCTGGGACATGCTCAACTCTTCTTCCAAGGAGGCCGACGCCAACGGCAATGCCAAGGGGAAGTAG

>Bradi5g24850_RGP4
ATGTCTTTGGAAGTTCAAGATAGCGAGGTTGACATTGTGATTGCAGCACTCCAGCCCAACCTGACCTCTTTCTTTGAGGCATGGCAGCCATTTTTCT
CCCGATTCCATATCATTGTTGTCAAAGATCCAGACATGGCAGCAGATCTTAAGATCCCTTCAGGTTTTGATGTCAAGGTTTACACAAAGTCAGACAT
TGAAGGATTGCTTGGTGCCACGTCCATCAACTTCTCTGGCCATTCATGCCGCTACTTTGGGTACCTTGTCTCACGCAAGAAGTATGTCATCTCAATT
GACGACAACTGCCTCCCGGCGAAGGACACCGCTGGGATGACTGTTGATGCTGTTACACAGCATATGATCAATTTGAAGACACCTGCTACACCTTTCT
TCTTCAACACACTATATGATCCATACCGCAAGGGGGCTGACTTTGTCCGTGGATACCCATTTAGCTTGCGTGAGGGGGTTGAATGCATGCTCTCATC
TGGCTTGTGGCTGCACAATGCCGACTAcGACCCAATGACACATGTCGTGAAGCGGAACCAACGCAACACAAATTATGTGGATGCTGTCATGACAGTT
CCACTTGGTGCGATGATGCCCGTGAGCGGGATAAATGTtGCATTCAACCGGGAGGTTCTGGGCCCCGTGATGTTCCCTGGCCTCCGGATACGCAAGG
AAGGGAAGCACAGATGGGATACCCTTGAAGACATATGGAATGGCTTGTGTGCCAAGGTgGTCTGCGACAGCTTAGGGTAtGGCGTGAAGACTGGACT
GCCTTATGTGATGAGGAGTGATGCAGAGGCAGGCAAAGCCCTGGAGAGCCTGAAAGATTGGGAAGGGGTGAAAGTGATGGACgATGTCCTTCCCTTC
TTCCAGTCGCTCAAGCTGTCGAGGACCGCGGTTACCGTCGACGACTGTGTTAAGGAGCTAGCAAGCATCGTGAGGGAGAAACTGGCACCGAAGAATC
CAATCTTCGCCAAAGCTGCTGATGCCATGGAGGAATGGACTAAACTCTGGAAAAGTCATGGAGCTCAGAATGCCTAG

>Bradi2g61940_AXS
tgaaccccaaatccagcaccaaatcccaaatccccccgcccccacgcgcattcccgtccccggatccggcggggagcATGTCGTCGTCGCCGGGGA
GCCTCGGCAGGACGGATCTGGACGGGAACCCCGTGGCGCCGCTCACCATCTGCATGATCGGCGCCGGCGGCTTCATTGGCTCCCACCTCTGCGAGAA
GCTCATGGCGGAGACGGCGCACGTCGTCCTCGCCGTCGACGTCTACTGCGACAAGATCCGCCACCTCGTCGACCCGGCCCCGCCCCACCTCTCCGGC
CGCATCTCCTTCCACCGCCTCAACATCAAGAACGACTCACGCCTCGAGGGCCTCATCAAGATGGCCGATCTGACGATCAACTTGGCGGCGATCTGCA
CGCCGGCGGACTACAACACGCGCCCGCTCGACACCATCTACAGCAACTTCATCGACGCCCTCCCAGTGGTCAAGTATTGCTCGGAGAACAACAAGCG
CCTCATCCACTTCTCCACGTGTGAGGTCTACGGCAAGACCATCGGCAGCTTCCTCCCCAAGGATCACCCGCTCCGCAAGGAAGCTGAATTTTATGTG
CTGAAAGAAGATGAGTCACCCTGCATTTTTGGTCCAATTGTGAAGCAGAGATGGTCCTACGCATGCGCGAAGCAGCTTATTGAGAGGCTTATTTTTG
CTGAAGGTGCAGAAAATGACCTTGAATTCACGATCGTGAGACCTTTCAATTGGATTGGGCCAAGGATGGACTTCATTCCTGGAGTTGATGGTCCTAG
CGAGGGTGTTCCTCGGGTTTTGGCTTGCTTCAGTAACAATCTCCTCCGCAGAGAGCCCCTGAAGCTTGTTGATGGCGGCGAGTCCCAGAGAACTTTC
GTTTACATCAAGGATGCCATTGAAGCTGTTCATTTGATGATTGAAAACCCTGCTCGAGCCAACGGCCATATCTTCAATGTTGGGAACCCCGACAATG
AAGTCACCGTTAGGGAATTGGCTGAAtTGATGATAGAGGTCTATGCTAATGTCTCAGGAGAGCCACCACTGGATGAACCTTTAATCGACGTGAGTGC
GAAGCAATTCTATGGTGAAGGATACGATGATAGTGACAAGAGAATTCCCGATATGACCCTAATCAACAAGCAGCTAGGGTGGAACCCAAAGACCCCT
CTCAAGGACTTGCTGGAGACGACTTTGACCTACCAGCACAAGACATACAAAGAAGCCGTCAAAAGGCAAATGTCACAGGCGACAGCGTCGAGTTAGa
aggaacatgtcgcgattccggatgcggcgatatatgttacaagcttatgtagagtcacggtcacagcggttggttgtgatctttcgcaaatctggat
acgcatactctagtatgatatttttgcgcctcttagagttactccctagatccctttttatcttaccctagattccgcgtgccgtgtaacggggca
aagcaaaggggattacctatatacctgtggcagagttgatgtattccattatacgatttggtagcacctcccgtgccattctttcctgctggtgaga
gctgttcttgttcagcatagatacatggtttcgatctcagttctgtcagttttagcacatagtctctttggttcctttgtcc

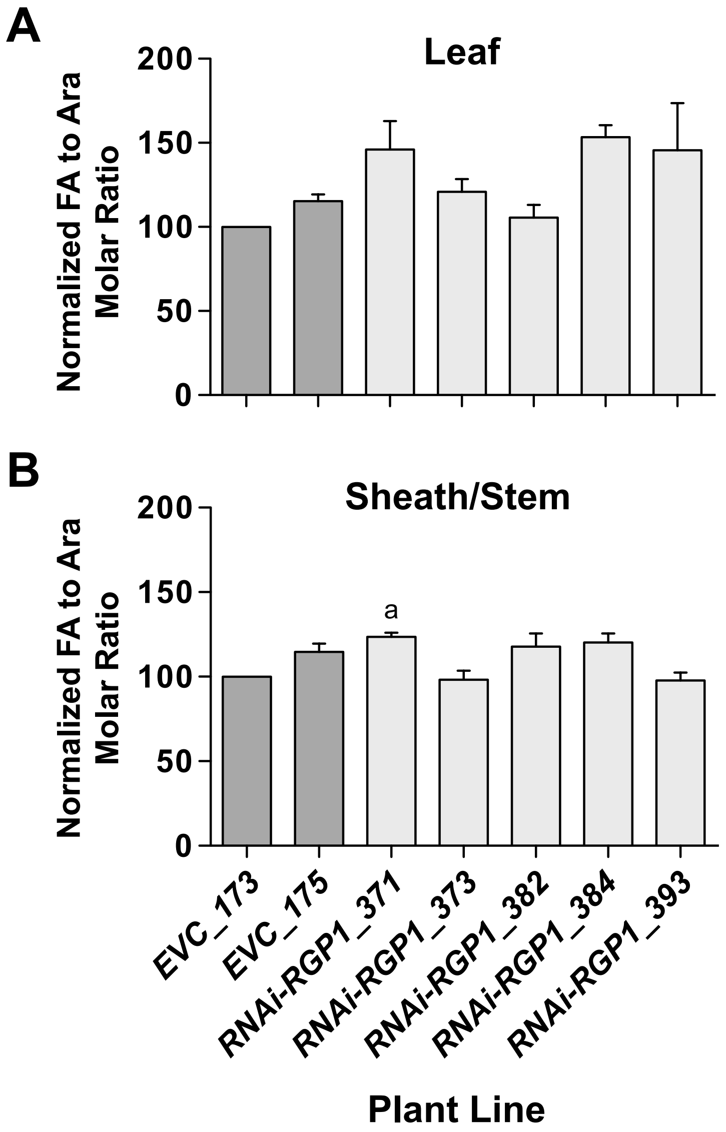

Supplemental Figure S8. FA to Ara ratios

(A) Leaf blade and (B) leaf sheath/stem normalized cell wall FA to Ara molar ratios from tissues of T_1 transgenic *Brachypodium empty-vector* control lines (*173* and *175*) and *RNAi-RGP1* lines (*371*, *373*, *382*, *384*, and *393*). All values were normalized with EVC_173 values set to 100%. The values for *EVC_173* were 0.113±0.012 and 0.243±0.007, respectively for leaf and sheath/stems. Error bars indicate SEM. a significantly different from *EVC_173* value (ANOVA with *post hoc* Tukey test, alpha=0.05).

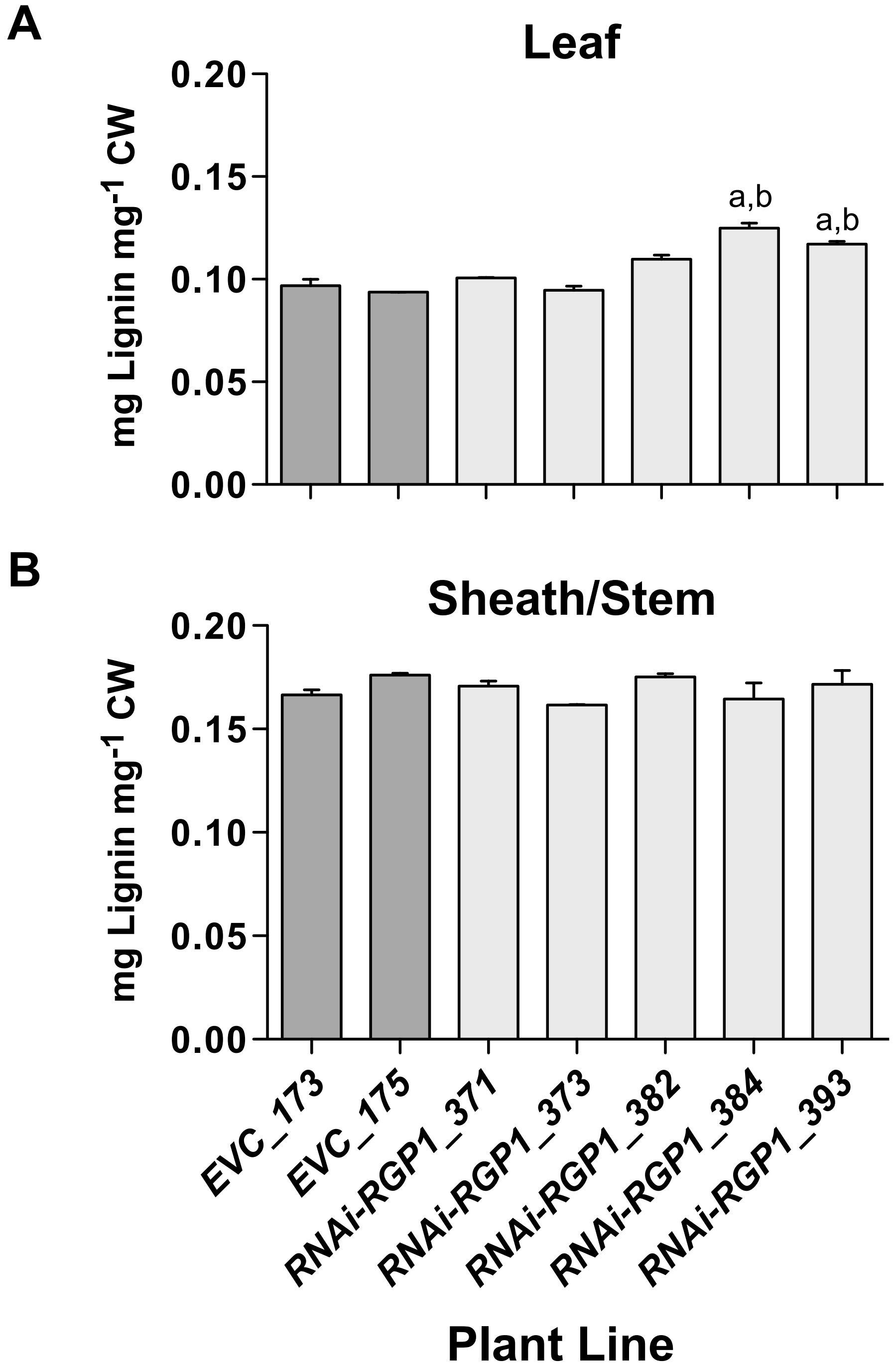

Supplemental Figure S9. Cell wall lignin
(A) Leaf blade and (B) leaf sheath/stem cell wall lignin concentrations from tissues of T_1 transgenic *Brachypodium* empty-vector control lines (*173* and *175*) and *RNAi-RGP1* lines (*371*, *373*, *382*, *384*, and *393*) were determined according to the acetyl bromide method. Error bars indicate SEM. [a,b] significantly different from *EVC_173* and *EVC_175* values, respectively (ANOVA with *post hoc* Tukey test, alpha=0.05).
